# Disease gene prioritization by integrating tissue-specific molecular networks using a robust multi-network model

**DOI:** 10.1186/s12859-016-1317-x

**Published:** 2016-11-10

**Authors:** Jingchao Ni, Mehmet Koyuturk, Hanghang Tong, Jonathan Haines, Rong Xu, Xiang Zhang

**Affiliations:** 1 Department of Electrical Engineering and Computer Science, Case Western Reserve University, 10900 Euclid Avenue, Cleveland, 44106 OH USA; 2 School of Computing, Informatics, Decision Systems Engineering, Arizona State University, 699 S. Mill Ave., Tempe, 85281 AZ USA; 3 Department of Epidemiology and Biostatistics, Case Western Reserve University, 10900 Euclid Avenue, Cleveland, 44106 OH USA; 4 College of Information Sciences and Technology, Pennsylvania State University, 332 Information Sciences and Technology Building, University Park, 16802 PA USA

**Keywords:** Disease gene prioritization, Tissue-specific molecular networks, Network of networks

## Abstract

**Background:**

Accurately prioritizing candidate disease genes is an important and challenging problem. Various network-based methods have been developed to predict potential disease genes by utilizing the disease similarity network and molecular networks such as protein interaction or gene co-expression networks. Although successful, a common limitation of the existing methods is that they assume all diseases share the same molecular network and a single generic molecular network is used to predict candidate genes for all diseases. However, different diseases tend to manifest in different tissues, and the molecular networks in different tissues are usually different. An ideal method should be able to incorporate tissue-specific molecular networks for different diseases.

**Results:**

In this paper, we develop a robust and flexible method to integrate tissue-specific molecular networks for disease gene prioritization. Our method allows each disease to have its own tissue-specific network(s). We formulate the problem of candidate gene prioritization as an optimization problem based on network propagation. When there are multiple tissue-specific networks available for a disease, our method can automatically infer the relative importance of each tissue-specific network. Thus it is robust to the noisy and incomplete network data. To solve the optimization problem, we develop fast algorithms which have linear time complexities in the number of nodes in the molecular networks. We also provide rigorous theoretical foundations for our algorithms in terms of their optimality and convergence properties. Extensive experimental results show that our method can significantly improve the accuracy of candidate gene prioritization compared with the state-of-the-art methods.

**Conclusions:**

In our experiments, we compare our methods with 7 popular network-based disease gene prioritization algorithms on diseases from Online Mendelian Inheritance in Man (OMIM) database. The experimental results demonstrate that our methods recover true associations more accurately than other methods in terms of AUC values, and the performance differences are significant (with paired *t*-test *p*-values less than 0.05). This validates the importance to integrate tissue-specific molecular networks for studying disease gene prioritization and show the superiority of our network models and ranking algorithms toward this purpose. The source code and datasets are available at http://nijingchao.github.io/CRstar/.

**Electronic supplementary material:**

The online version of this article (doi:10.1186/s12859-016-1317-x) contains supplementary material, which is available to authorized users.

## Background

Identifying disease-causing genes is a fundamental challenge in human health. Traditional linkage mapping or more recent genome-wide association studies aim to identify genomic intervals that contain disease causal genes [1]. The identified intervals typically contain tens to hundreds of disease-gene candidates, but identifying the particular gene and causal mutation remains difficult because experimentally validating a large amount of disease-gene candidates is expensive. Therefore, it is important to design efficient methods to prioritize disease-gene candidates. Recently, a series of sophisticated network-based computational methods have been developed to predict the most promising disease genes. The common motivation of these methods is that genes causing the same or similar diseases tend to lie close to one another in the molecular networks [2–6].

**Fig. 1:**
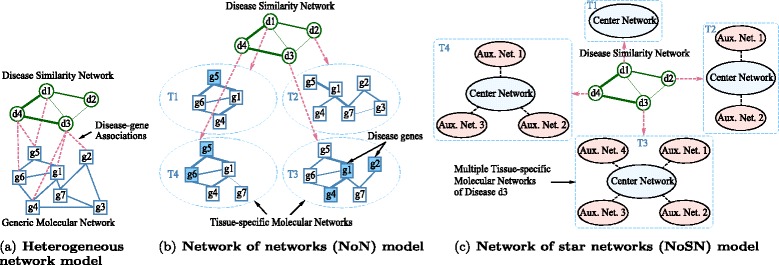
Different network models for disease gene prioritization. **a** the traditional heterogeneous network model, **b** the network of networks (NoN) model, where T1 to T4 represent different tissues (and their specific molecular networks) that are specific to the corresponding diseases, and **c** the network of star networks (NoSN) model, where each disease corresponds to multiple molecular networks of its specific tissue. In the NoN and NoSN models, the known disease-gene associations are regarded as the seed nodes. In **b**, the seed nodes are highlighted in *blue*

Figure 1(a) shows the typical network model used by most of the existing methods. There are three components in this model: a disease network representing the similarities between different diseases, a molecular network showing the interactions or functional associations between molecules, such as the protein-protein interaction network (PPIN) or the gene co-expression network (GCN), and known disease-gene associations linking diseases and molecules. Such a network model is usually referred to as the *heterogeneous network model* because of the heterogeneity between the disease network and molecular network [7]. Based on this heterogeneous network model, various approaches have been proposed, including regression [8], network alignment [9], random walk [7, 10], maximum flow [11], label propagation [12, 13], and supervised link prediction [10, 14].

A limitation of this heterogeneous network model is that it uses a single generic molecular network to predict genes for all different diseases. It implicitly assumes that all diseases share exactly the same molecular network. However, recent studies have shown that the majority of genetic disorders manifest only in a single or a few tissues [15–17], and the molecular networks in different tissues are usually different [18–20]. For example, Bossi et al. [18] analyzed human protein interactions and found that proteins have tissue-specific roles and form tissue-specific interactions. Furthermore, Lage et al. [21] and Magger et al. [16] found that the majority of the known disease genes are significantly expressed in the tissues where the corresponding diseases manifest, thus gene expression is also tissue specific. Therefore, instead of using a generic molecular network for all diseases, an ideal method should take tissue-specificity into consideration.

So far, limited research has been done to integrate tissue-specific networks for disease gene prioritization. The most relevant work to us is the work by Magger et al. [16]. In that paper, for each query disease, its corresponding tissue-specific PPIN was used to replace the generic molecular network in the heterogeneous network model. It had been shown to be more accurate than using a single generic molecular network. The limitation of this approach is that even though the molecular network may not be relevant to other diseases, it is still shared by all diseases.

To address the limitation of the existing methods, in this paper, we develop a more robust and flexible network model [22–24]. In this model, each disease is allowed to have its own tissue-specific molecular network. An example is shown in Fig. 1(b). In this figure, there are four tissue-specific molecular networks, each for a disease in the disease similarity network. The known disease genes are highlighted in blue. This network model can be treated as a (disease) network of (tissue-specific molecular) networks. We refer to such a model as a network of networks (NoN). Compared to the heterogeneous network model shown in Fig. 1(a), a distinct advantage of the NoN model is that it does not require all diseases to share the same generic molecular network.

The NoN model in Fig. 1(b) allows one tissue-specific molecular network for each disease. In practice, multiple or different types of tissue-specific networks may be available, such as tissue-specific PPINs [16] and tissue-specific GCNs [25]. These networks provide complementary information about the diseases. To incorporate multiple tissue-specific molecular networks, we further extend the basic NoN model to the network of star networks (NoSN) model. An example is shown in Fig. 1(c). In this figure, for each disease, there is a center network (corresponding to the single tissue-specific molecular network in the basic NoN model) and a set of auxiliary networks. These auxiliary networks provide extra information of the diseases and can be utilized to further improve the accuracy of candidate gene prediction.

We formulate disease gene prioritization as optimization problems based on network propagation techniques. In particular, the known disease genes are used as seeds. The ranking scores of different genes are (1) smooth within each molecular network, (2) biased toward the seed genes, and (3) consistent in molecular networks of similar diseases. To solve the problems, we develop a family of novel algorithms which generalize single network propagation algorithm to NoN and NoSN. Our algorithms are fast with almost linear time complexity w.r.t. the network sizes. We also provide rigorous theoretical foundations for our algorithms in terms of their optimality and convergence properties. Another novelty is that when multiple tissue-specific networks are available for a disease, our method can automatically infer the relative importances of different networks, thus is robust to noisy and incomplete networks.

We refer to the gene prioritization problem based on NoN as CrossRank, and the problem based on NoSN as CrossRankStar, which will be discussed in the following sections.

## Methods

### CrossRank - candidate gene prioritization on NoN

We first discuss how to formulate the disease gene prioritization problem as an optimization problem using the basic NoN model and introduce CrossRank. Our problem formulation generalizes the existing label propagation methods designed for using a single generic molecular network [12, 13] to NoN. Important symbols used in this paper are summarized in Table 1.

**Table 1:** Summary of symbols

Symbol	definition
**A**	The adjacency matrix of disease similarity network
**G**_*i*_	The adjacency matrix of the tissue-specific molecular network of
	disease *i* (for NoN)
**G**_*i*∗_	The adjacency matrix of the center molecular network of disease *i*
	(for NoSN)
**G**_*ip*_	The adjacency matrix of the *p* ^th^ auxiliary molecular network of
	disease *i* (for NoSN)
**I**_*n*_	An *n*×*n* identity matrix
**r**_*i*_	The ranking score vector of genes in **G**_*i*_ (for NoN)
**e**_*i*_	The seed vector of genes in **G**_*i*_ (for NoN)
**r**_*i*∗_	The ranking score vector of genes in **G**_*i*∗_ (for NoSN)
**e**_*i*∗_	The seed vector of genes in **G**_*i*∗_ (for NoSN)
**r**_*ip*_	The ranking score vector of genes in **G**_*ip*_ (for NoSN)
**e**_*ip*_	The seed vector of genes in **G**_*ip*_ (for NoSN)
*h*	Number of diseases in **A**
*n*_*i*_	Number of genes in **G**_*i*_ (for NoN)
*n*_*i*∗_	Number of genes in **G**_*i*∗_ (for NoSN)
*n*_*ip*_	Number of genes in **G**_*ip*_ (for NoSN)
*k*_*i*_	Number of auxiliary molecular networks of disease *i* (for NoSN)
*d*_**A**_(*i*)	Degree of disease *i* in **A**, i.e., $d_{\mathbf {A}}(i) = \sum _{j=1}^{h}\mathbf {A}(i,j)$
$\mathcal {I}_{ij}$	The set of common genes in **G**_*i*_ and **G**_*j*_ (for NoN)
$\bar {\mathcal {I}}_{ij}$	The set of genes in **G**_*i*_ but not in **G**_*j*_ (for NoN)
$\mathcal {I}_{i*,j*}$	The set of common genes in **G**_*i*∗_ and **G**_*j*∗_ (for NoSN)
$\bar {\mathcal {I}}_{i*,j*}$	The set of genes in **G**_*i*∗_ but not in **G**_*j*∗_ (for NoSN)
$\mathcal {I}_{i*,ip}$	The set of common genes in **G**_*i*∗_ and **G**_*ip*_ (for NoSN)
$\bar {\mathcal {I}}_{i*,ip}$	The set of genes in **G**_*i*∗_ but not in **G**_*ip*_ (for NoSN)

Suppose that there are *h* diseases in the disease similarity network and let **A** be its adjacency matrix. Thus **A**(*i*,*j*) measures the similarity between diseases *i* and *j* (1≤*i*,*j*≤*h*). For disease *i*, suppose there are *n*_*i*_ genes in its tissue-specific molecular network and let **G**_*i*_ be the adjacency matrix of the tissue-specific molecular network (note different **G**_*i*_ may be built on different sets of genes). We use vector **r**_*i*_ to represent the ranking scores of genes in molecular network **G**_*i*_.

The known disease genes are used as seed nodes. We denote the seed vector in **G**_*i*_ as **e**_*i*_: if *x* is a seed node, then $\mathbf {e}_{i}(x) = \frac {1}{s_{i}}$, where *s*_*i*_ is the total number of seeds in **G**_*i*_; otherwise **e**_*i*_(*x*)=0. If there is no known disease genes, then **e**_*i*_=**0**.

There are three criteria in our problem formulation. The first two criteria focus on ranking scores within individual molecular networks. They are commonly used in previous network prorogation methods [12, 13, 26]. The third criterion is specific to the NoN model. Next, we introduce them one by one.

The first criterion is the *within-network smoothness*, i.e., the ranking scores of nearby genes in a molecular network should be smooth. That is, (**r**_*i*_(*x*)−**r**_*i*_(*y*))^2^ **G**_*i*_(*x*,*y*) should be as small as possible. In matrix form, this term can be represented as $\mathbf {r}_{i}^{T}(\mathbf {I}_{n_{i}} - \tilde {\mathbf {G}}_{i})\mathbf {r}_{i}$, where $\tilde {\mathbf {G}}_{i}$ is the symmetrically normalized adjacency matrix of **G**_*i*_, and $\mathbf {I}_{n_{i}}$ is an identity matrix of size *n*_*i*_×*n*_*i*_.

The second criterion is the *within-network seed preference*, i.e., the ranking scores of genes in a molecular network should favor the known disease genes. That is, $\|\mathbf {r}_{i} - \mathbf {e}_{i}\|_{F}^{2}$ should be as small as possible, where $\|\cdot \|_{F}^{2}$ is the Frobenius norm.

Putting these two criteria together, we have the following objective function to measure the within-network smoothness and seed preference of the gene ranking scores

1 $$ \begin{aligned} &\Theta_{\text{within}}(\mathbf{r}_{i}) = c{\mathbf{r}_{i}^{T}(\mathbf{I}_{n_{i}} - \tilde{\mathbf{G}}_{i})\mathbf{r}_{i}} + (1-c){\|\mathbf{r}_{i} - \mathbf{e}_{i}\|_{F}^{2}} \end{aligned}  $$

where *c* is a regularization parameter balancing the weights of these two terms.

The third criterion in our objective function is the *cross-network consistency*: if diseases *i* and *j* are highly similar, i.e., high **A**(*i*,*j*) value, their common genes should have similar rankings in their corresponding molecular networks.

More formally, let $\mathcal {I}_{ij}$ be the set of common genes shared by molecular networks **G**_*i*_ and **G**_*j*_. Let $\mathbf {r}_{i}(\mathcal {I}_{ij})$ and $\mathbf {r}_{j}(\mathcal {I}_{ij})$ be the ranking scores of the common genes in **G**_*i*_ and **G**_*j*_ respectively. The difference between $\mathbf {r}_{i}(\mathcal {I}_{ij})$ and $\mathbf {r}_{j}(\mathcal {I}_{ij})$ should be small for a large similarity value **A**(*i*,*j*) between diseases *i* and *j*. This is because similar diseases tend to have similar disease genes. That is, we want to minimize

$$\begin{aligned} \mathbf{A}(i,j)\left[{\left\|\frac{\mathbf{r}_{i}(\mathcal{I}_{ij})}{\sqrt{d_{\mathbf{A}}(i)}}-\frac{\mathbf{r}_{j}(\mathcal{I}_{ij})}{\sqrt{d_{\mathbf{A}}(j)}}\right\|_{F}^{2}} \right] \end{aligned} $$

where *d*_**A**_(*i*) and *d*_**A**_(*j*) represent the degrees of diseases *i* and *j* in the disease similarity network, respectively. In this paper, we define the degree of a node *i* as the sum of edge weights incident on it, i.e., $d_{\mathbf {A}}(i) = \sum _{j=1}^{h}\mathbf {A}(i,j)$. In the above equation, we normalize ranking scores $\mathbf {r}_{i}(\mathcal {I}_{ij})$ and $\mathbf {r}_{j}(\mathcal {I}_{ij})$ by the degrees of diseases *i* and *j* to make them comparable for different diseases.

In addition to penalizing the difference between the common gene ranking scores in different networks, we also penalize the ranking scores of the genes not in common. If a gene exists in **G**_*i*_ but not in **G**_*j*_, this indicates it is not highly expressed in the relevant tissue of disease *j*. Then we regard it as having a zero score in **G**_*j*_. This will decrease its score in **G**_*i*_ as well, since it is less likely to be a disease gene than genes that are highly expressed in both relevant tissues of diseases *i* and *j*. Putting these two aspects together, we have the following criterion to measure the cross-network consistency of the gene ranking scores

2 $$ \begin{aligned} \Theta_{\text{cross}}(\mathbf{r}_{i}, \mathbf{r}_{j}) =~&\mathbf{A}(i,j) \left[{\left\|\frac{\mathbf{r}_{i}(\mathcal{I}_{ij})}{\sqrt{d_{\mathbf{A}}(i)}}-\frac{\mathbf{r}_{j}(\mathcal{I}_{ij})}{\sqrt{d_{\mathbf{A}}(j)}}\right\|_{F}^{2}}\right.\\ &\!\!\left.+ \left\|\frac{\mathbf{r}_{i}(\bar{\mathcal{I}}_{ij})}{\sqrt{d_{\mathbf{A}}(i)}}\|_{F}^{2} + \|\frac{\mathbf{r}_{j}(\bar{\mathcal{I}}_{ji})}{\sqrt{d_{\mathbf{A}}(j)}}\right\|_{F}^{2} \right] \end{aligned}  $$

where $\bar {\mathcal {I}}_{ij}$ ($\bar {\mathcal {I}}_{ji}$) represents the set of genes in **G**_*i*_ (**G**_*j*_) but not in **G**_*j*_ (**G**_*i*_).

Integrating Eqs. (1, 2), we have the overall objective function as follows

3 $$ \begin{aligned} \mathrm{J}_{\text{CR}} = \sum\limits_{i=1}^{h}\Theta_{\text{within}}(\mathbf{r}_{i}) + \beta \sum\limits_{i,\,j=1}^{h}\Theta_{\text{cross}}(\mathbf{r}_{i}, \mathbf{r}_{j}) \end{aligned}  $$

where *β* is a regularization parameter that controls the importance of the second term.

Note that the well known label propagation methods [12, 13, 26] only optimize the within-network smoothness and seed preference criteria for a single network, i.e., *Θ*_within_. In our method, we generalize it to multiple networks and further introduce the cross-network consistency criterion *Θ*_cross_.

### CrossRankStar - candidate gene prioritization on NoSN

To allow each disease to have multiple tissue-specific molecular networks, in the following, we introduce CrossRankStar, which formulates the disease gene prioritization problem based on NoSN shown in Fig. 1(c). In NoSN, each disease has a center molecular network. This center molecular network has the highest quality among all available tissue-specific molecular networks for that disease. Other molecular networks are used as auxiliary networks around the center network. In practice, the center network can be selected by domain knowledge, or by the reliabilities of different data types. For example, in our experiments, tissue-specific GCNs are generally more noisy than tissue-specific PPINs, thus the tissue-specific PPINs are more reliable than the tissue-specific GCNs.

We use **G**_*i*∗_ to represent the adjacency matrix of the center network of disease *i*, and **G**_*ip*_ to represent the adjacency matrix of the *p* ^th^(1≤*p*≤*k*_*i*_) auxiliary network of disease *i*, where *k*_*i*_ is the number of auxiliary networks of disease *i*. Similarly, **r**_*i*∗_ and **r**_*ip*_ represent the ranking score vectors of genes in **G**_*i*∗_ and **G**_*ip*_, respectively. **e**_*i*∗_ and **e**_*ip*_ represent the seed vectors of genes in **G**_*i*∗_ and **G**_*ip*_, respectively.

Similar as before in CrossRank, in CrossRankStar we also have the *within-network smoothness* and *within-network seed preference* criteria for all molecular networks. Thus the within network objective functions *Φ*_within_(**r**_*i*∗_) and *Φ*_within_(**r**_*ip*_) can be defined in a similar way as *Θ*_within_(**r**_*i*_) in Eq. (1): we can simply replace the subscript *i* in Eq. (1) by *i*∗ (and *ip*), and get *Φ*_within_(**r**_*i*∗_) (and *Φ*_within_(**r**_*ip*_)).

In NoSN, the *cross-network consistency* criterion between diseases is applied to center networks. That is, if two diseases are highly similar, the common genes in their center networks should have consistent ranking scores. The cross-network objective function *Φ*_cross_(**r**_*i*∗_,**r**_*j*∗_) can be similarly defined as *Θ*_cross_(**r**_*i*_,**r**_*j*_) in Eq. (2) by replacing *i* and *j* in Eq. (2) by *i*∗ and *j*∗, respectively.

The criteria we have discussed so far are inherited from the previous model on NoN. In NoSN, since now we have multiple networks for each disease, we have another *cross-network consistency* constraint, i.e., the ranking scores of the same genes should be consistent in the networks of the same disease. This is done by penalizing the difference between the ranking vectors **r**_*i*∗_ and **r**_*ip*_, which can be defined as follows.

4 $$ \begin{aligned} \Phi_{\text{cross}}'(\mathbf{r}_{i*}, \mathbf{r}_{ip}) = &\left\| \frac{\mathbf{r}_{i*}\left(\mathcal{I}_{i*,ip}\right)}{\sqrt{k_{i}}} - \mathbf{r}_{ip}\left(\mathcal{I}_{i*,ip}\right) \right\|_{F}^{2}\\ &+ \left\| \frac{\mathbf{r}_{i*}\left(\bar{\mathcal{I}}_{i*,ip}\right)}{\sqrt{k_{i}}} \|_{F}^{2} + \| \mathbf{r}_{ip}\left(\bar{\mathcal{I}}_{ip,i*}\right) \right\|_{F}^{2} \end{aligned}  $$

where $\mathbf {r}_{i*}(\mathcal {I}_{i*,ip})$ ($\mathbf {r}_{ip}(\mathcal {I}_{i*,ip})$) represents the ranking scores of the common genes in **G**_*i*∗_ (**G**_*ip*_), and $\bar {\mathcal {I}}_{i*,ip}$ ($\bar {\mathcal {I}}_{ip,i*}$) represents the set of genes in **G**_*i*∗_ (**G**_*ip*_) but not in **G**_*ip*_ (**G**_*i*∗_). Eq. (4) is the cross-network consistency criterion applied to the networks of the same disease. Note that we normalize ranking vector **r**_*i*∗_ by its degree *k*_*i*_ to make it comparable to **r**_*ip*_.

Integrating *Φ*_within_(**r**_*i*∗_), *Φ*_within_(**r**_*ip*_), *Φ*_cross_(**r**_*i*∗_,**r**_*j*∗_) and *Φ*cross′(**r**_*i*∗_,**r**_*ip*_), we obtain the following objective function on the NoSN

5 $$ \begin{aligned} \mathrm{J}_{\text{CRstar}} = &\sum\limits_{i=1}^{h}\left(\Phi_{\text{within}}(\mathbf{r}_{i*}) + \sum\limits_{p=1}^{k_{i}}\Phi_{\text{within}}(\mathbf{r}_{ip})\right)\\ &+ \alpha \sum\limits_{i=1}^{h}\sum\limits_{p=1}^{k_{i}}\Phi_{\text{cross}}'(\mathbf{r}_{i*}, \mathbf{r}_{ip})\\ &+ \beta \sum\limits_{i,j=1}^{h}\Phi_{\text{cross}}(\mathbf{r}_{i*}, \mathbf{r}_{j*}) \end{aligned}  $$

where *α* and *β* are two regularization parameters balancing the weights of the two corresponding terms.

Comparing to the objective function J_CR_ in Eq. (3), the major difference of J_CRstar_ in Eq. (5) is the consideration of the auxiliary networks in *Φ*_within_(**r**_*ip*_) and *Φ*cross′(**r**_*i*∗_,**r**_*ip*_).

### Weighted CrossRankStar

The optimization problem of J_CRstar_ in Eq. (5) treats all auxiliary networks equally for a disease. In practice, different tissue-specific molecular networks may have different qualities since some networks may contain more noises or be more incomplete than others. Therefore, an ideal method should be able to automatically determine the relative importances of these auxiliary networks.

To achieve this, we modify Eq. (5) by assigning a weight *α*_*ip*_ (*α*_*ip*_≥0) to the ranking inconsistency term *Φ*cross′(**r**_*i*∗_,**r**_*ip*_), and learn *α*_*ip*_ automatically. Intuitively, the larger the ranking inconsistency *Φ*cross′(**r**_*i*∗_,**r**_*ip*_), the smaller the weight *α*_*ip*_. Let $\phantom {\dot {i}\!}\boldsymbol {\alpha }_{i} = (\alpha _{i1},..., \alpha _{{ik}_{i}})^{T}$ be the column vector of the weights for disease *i*. We require $\sum _{p=1}^{k_{i}}\alpha _{ip} = 1$ such that the weights in ***α***_*i*_ are comparable for different diseases. Therefore, the weighted version of CrossRankStar is

6 $$ \begin{aligned} \mathrm{J}_{\text{WCRstar}} = &\sum\limits_{i=1}^{h}\left(\Phi_{\text{within}}(\mathbf{r}_{i*}) + \sum\limits_{p=1}^{k_{i}}\Phi_{\text{within}}(\mathbf{r}_{ip})\right)\\ &+ \sum\limits_{i=1}^{h}\sum\limits_{p=1}^{k_{i}}\alpha_{ip}\Phi_{\text{cross}}'(\mathbf{r}_{i*}, \mathbf{r}_{ip})\\ &+ \beta \sum\limits_{i,j=1}^{h}\Phi_{\text{cross}}(\mathbf{r}_{i*}, \mathbf{r}_{j*}) + \gamma \sum\limits_{i=1}^{h}\left\|\boldsymbol{\alpha}_{i}\right\|_{F}^{2}\\ \end{aligned}  $$

In the last term of the above equation, we use *ℓ*_2_-norm regularization on ***α***_*i*_ so that we can control non-zero weights in ***α***_*i*_ by varying the parameter *γ* (*γ*>0). This is useful and can help avoid trivial solutions. Without it, all weights in ***α***_*i*_ will be zero except for the one with the least inconsistency *Φ*cross′(**r**_*i*∗_,**r**_*ip*_). This overfitting prevents the use of other informative auxiliary networks. By setting a larger value to *γ*, the more weights in ***α***_*i*_ will be assigned non-zero values. A mathematical discussion about this based on the optimization solution can be found in the Additional file 1 (Sec. Optimization Solution to J_WCRstar_). This is also verified in our experimental results in the Additional file 2 (Sec. Selectivity of Parameter *γ* of WCRSTAR).

### Optimization methods

#### Solutions to CrossRank and CrossRankStar

The detailed techniques for minimizing the objective functions J_CR_ in Eq. (3) and J_CRstar_ in Eq. (5) can be found in the Additional file 3 and the Additional file 4, respectively. Here, we give a brief overview. The objective function J_CR_ in Eq. (3) is jointly convex in **r**_1_,...,**r**_*h*_. This can be shown by first deriving its matrix form, which is a quadratic function of $\mathbf {r} = (\mathbf {r}_{1}^{T},..., \mathbf {r}_{h}^{T})^{T}$ (i.e., the concatenated ranking score vector of all molecular networks). Similarly, the matrix form of J_CRstar_ in Eq. (5) is a quadratic function of a concatenated vector of {**r**_*i*∗_} and {**r**_*ip*_}. Their matrix forms can be found in Eq. (3) in the Additional file 3 and Eq. (4) in the Additional file 4, respectively, from which we derive power methods to minimize J_CR_ and J_CRstar_, i.e., Eq. (5) in the Additional file 3 and Eq. (6) in the Additional file 4. These equations are fixed-point updating rules to compute the concatenated vectors **r**’s that converge to the global optimal solutions of J_CR_ and J_CRstar_, respectively. They have the similar form to the label propagation methods [12] and thus are easy to implement.

The detailed algorithms are included in the Additional file 3 as Algorithm CR and in the Additional file 4 as Algorithm CRSTAR, which are efficient with almost linear time and space complexities. After the algorithms converge, we can break **r** down into {**r**_*i*_} (for CR) or {**r**_*i*∗_} and {**r**_*ip*_} (for CRSTAR) and rank genes in each molecular network by their scores. The theoretical analysis of the complexity, convergence and optimality of CR and CRSTAR can also be found in the Additional file 3 and the Additional file 4, respectively.

#### Solution to weighted CrossRankStar

The objective function J_WCRstar_ in Eq. (6) is not jointly convex. Therefore, we minimize Eq. (6) by an alternating minimization approach, i.e., the objective function is alternately minimized with respect to one variable while fixing others. This procedure repeats until convergence. Specifically, we solve **r**_*i*∗_ and **r**_*ip*_ according to Theorem 1 and Theorem 2 in the Additional file 1, respectively. We solve ***α***_*i*_ using a method derived from the Karush-Kuhn-Tucker (KKT) conditions [27], which is similar to the method in [28]. The details of solving ***α***_*i*_ are included in the Additional file 1 (Sec. Optimization Solution to J_WCRstar_).

Since each of the updating strategies for **r**_*i*∗_, **r**_*ip*_ and ***α***_*i*_ decreases the value of the objective function J_WCRstar_ in Eq. (6), and J_WCRstar_ is lower bounded by 0, alternately updating **r**_*i*∗_, **r**_*ip*_ and ***α***_*i*_ will converge. The detailed algorithm is summarized in the Additional file 1 as Algorithm WCRSTAR, which is efficient with almost linear time and space complexities. The complexity analysis of Algorithm WCRSTAR can also be found in the Additional file 1.

### Data sources

In this section, we describe the datasets that will be used to evaluate our methods.

#### Disease similarity network

We use the frequently used disease similarity network from [29], which contains 5080 diseases. The similarities are calculated based on the medical subject headings description in the Online Mendelian Inheritance in Man (OMIM) database [30]. Following the approach in [7, 11, 31], we construct a *k*-nearest-neighbor graph of the disease similarity network with *k*=5, a good choice that has been evaluated by earlier studies [7, 11, 31]. By doing so, there are 21006 edges in the disease similarity network.

#### Disease-tissue mapping matrix

To map diseases to tissues, we use the disease-tissue association matrix from [21]. In this matrix, each tissue is assigned to a disease with certain probability. The probability between a disease and a tissue is estimated according to their co-occurrences in the PubMed abstracts. The matrix contains association probabilities between 965 diseases and 68 tissues.

#### Tissue-specific PPINs

The tissue-specific PPINs for 60 tissues are obtained from [16]. They are constructed by removing lowly expressed genes w.r.t. each tissue from a generic PPIN of 9998 proteins. Magger et al. [16] considered a gene as lowly expressed in a tissue if its expression in that tissue was below 200 Affymetrix average-difference (AD) units. The number of nodes and edges in the generated tissue-specific PPINs range in [942,6702] and [2026,27571], respectively.

#### Tissue-specific GCNs

We use a recently published human gene expression dataset [25] to construct tissue-specific GCNs. In this dataset, 19 tissues also exist in the disease-tissue mapping matrix, thus can be used in our experiments. These 19 tissue-specific GCNs are generated by first calculating the Pearson correlation coefficients between the expression profiles of tissue-specific genes, which range in [−1,1]. We normalize the correlation coefficients to range in [0,1] by using the widely used Weighted Gene Co-expression Network Analysis (WGCNA) [32, 33]. Specifically, each correlation coefficient *c* *o* *r*(*x*,*y*) between two genes *x* and *y* is normalized to a similarity score *s*(*x*,*y*) by

$$s(x,y) = (0.5 + 0.5cor(x,y))^{\kappa} $$

where the power *κ* is a soft thresholding parameter. We use the typical setting of *κ*=12 in our experiments^1^. Finally, we constructed the *k*-nearest-neighbor graph with *k*=5 which generally gave more reliable results among *k*∈{3,5,10} in our experiments. The number of nodes and edges in these tissue-specific GCNs range in [985,1515] and [3506,5957], respectively.

#### Known disease-gene associations

We use two versions of disease-gene associations that are frequently used in previous studies [13, 31]. These two sets of associations are obtained from OMIM on May 2007 and May 2010, respectively. May-2007 version contains 1393 associations between 1126 diseases and 916 genes. May-2010 version contains 2187 associations between 1524 diseases and 1326 genes.

### Construction of NoN and NoSN

**Fig. 2:**
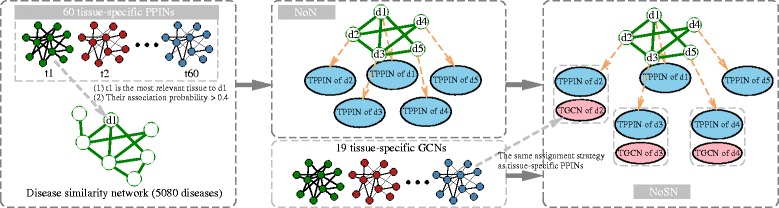
An illustration for NoN and NoSN construction. TPPIN: tissue-specific PPIN. TGCN: tissue-specific GCN. First, each disease in the disease similarity network is assigned a TPPIN using the disease-tissue association matrix, if the shown two criteria are satisfied. Thus we obtain an NoN. Then each disease in the NoN is assigned a TGCN as the auxiliary molecular network to form an NoSN, using the same strategy as assigning TPPINs to diseases. Please see text for details

Figure 2 illustrates the construction processes of NoN and NoSN. To construct NoN, we first assign the 60 tissue-specific PPINs to diseases. By mapping the diseases in the disease similarity network and those covered by the disease-tissue association matrix, 926 diseases have tissue associations. Using the disease-tissue association matrix, we assign a tissue-specific PPIN to a disease if the tissue is the most relevant tissue of the disease and their association probability is above 0.4 (as suggested in [16, 21]). This ensures that the considered diseases show strong tissue specificities. After this step, 361 diseases are assigned with tissue-specific PPINs, which covers 38.98 *%* diseases that have tissue associations.

To construct NoSN, we further assign the 19 tissue-specific GCNs to their corresponding diseases as auxiliary networks by using the disease-tissue association matrix. A disease may not have an auxiliary network if its most relevant tissue does not appear in the 19 tissues of GCNs.

## Results and discussion

In this section, we present comprehensive experimental results to evaluate the performance of our ranking algorithms CR, CRSTAR and WCRSTAR.

### Baseline methods

We compare our methods to both the classic and the state-of-the-art network-based algorithms. CIPHER-DN and CIPHER-SP [8] are classic regression based methods. They score a candidate gene for a query disease by the Pearson correlation coefficient between their respective similarities to all diseases. RWRH [7] is a random walk with restart algorithm on the heterogeneous network model. PRINCE [12] is a label propagation method derived from [26]. BIRW [13] alternately propagates labels on disease similarity network and molecular network with certain number of walks to reconstruct the association network. Katz [34] is popular for social network link prediction [35]. It is also shown to be effective for disease-gene association prediction in [10]. The authors of [10] also propose a supervised link prediction method CATAPULT. It extracts walk-based features from the network and learns a biased SVM model from the positive and unlabeled examples to predict unseen disease-gene associations. The authors of [10] show that CATAPULT performs better than another supervised link prediction method, ProDiGe [14]. We have tested ProDiGe on our datasets and get the same conclusion. The results of ProDiGe can be found in the Additional file 2 (Sec. More Results on Accuracy Evaluation). The parameters of these algorithms are tuned using leave-one-out cross validation for optimal performance. Note that all these methods are developed on the heterogeneous network model as shown in Fig. 1(a).

### Accuracy evaluation

We first evaluate the accuracy of the selected methods by leave-one-out cross validation which is a widely used method in many existing works [12, 16]. Each time, a known disease-gene association (*d*,*g*) is removed together with all other disease-gene associations involving *g*. The selected methods are assessed by their abilities to uncover the removed association (*d*,*g*) when querying *d*.

Since the heterogeneous network model is not designed to handle multiple tissue-specific molecular networks, each time when querying a disease, we use its most relevant tissue-specific molecular network to replace the generic molecular network and apply the baseline methods on the resulted heterogeneous network. Note that this approach has been shown to achieve better performance than using the same generic molecular network for all query diseases [16].

Table 2 shows the average AUC values (across all cross validation runs) of the compared methods. We report the AUC values for up to 50, 100, 300, 500, 700 and 1000 false positives. These values are effective to estimate the prediction accuracy of each method for top ranked genes and have been widely used to evaluate gene prioritization methods [13, 22, 31]. For example, the average AUC50 is large if many test genes are ranked highly among the top 50 of the ranking list and the average AUC50 is 1 if all test genes are ranked first in their respective validation runs. Note that the traditional AUC value calculates the area under the ROC curve over all false positives, which is not suitable in practice where only top ranked genes will be experimentally studied later in a usual disease gene identification process. Thus we only look at AUC values over certain number of highly ranked false positives to see if the true positives are discovered before these false positives.

**Table 2:** AUC value comparison

Network model	Method	AUC50	AUC100	AUC300	AUC500	AUC700	AUC1000
Heterogeneous network	CIPHER-DN	0.2332***	0.2439***	0.2510***	0.2524***	0.2530***	0.2535***
	CIPHER-SP	0.2068***	0.2478***	0.3112***	0.3369***	0.3568***	0.3790***
	RWRH	0.2382***	0.2849***	0.3849***	0.4503**	0.4922**	0.5388**
	PRINCE	0.2632*	0.3065*	0.3787**	0.4247***	0.4594***	0.5092***
	BIRW	0.2615*	0.3082*	0.4095*	0.4653	0.5068*	0.5513*
	Katz	0.2101***	0.2726***	0.3831**	0.4451*	0.4838**	0.5289**
	CATAPULT	0.1370***	0.1957***	0.3148***	0.3803***	0.4315***	0.4875***
NoN	CR	0.2711*	0.3235	0.4244	0.4815	0.5233	0.5665
NoSN ^a^	CRstar	***0.2900***	***0.3408***	***0.4347***	***0.4890***	***0.5331***	***0.5779***
NoSN ^b^	CRstar	0.2900	0.3400	0.4355	0.4882	0.5331	0.5798
	WCRstar	***0.2906***	***0.3409***	***0.4384***	***0.4973***	***0.5415***	***0.5863***

^a^NoSN with one set of tissue-specific GCNs. ^b^NoSN with two sets of tissue-specific GCNs. The *p*-value ranges: * represents 0.005∼0.05, ** represents 0.0005∼0.005, *** represents <0.0005

In the first two panels of Table 2 (i.e., network models Heterogeneous network, NoN and NoSN ^a^), we present the statistical significance of the paired *t*-test between the AUC values of CRSTAR and other methods. Specifically, for each setting, e.g., AUC100, every method has a vector of AUC values (with each entry being the AUC value for one test gene). The paired *t*-test is performed between the AUC vector generated by CRSTAR and the ones generated by other methods. In Table 2, we only report the ranges of the *p*-values represented by ∗’s. The exact *p*-values can be found in the Additional file 2 (Sec. The *p*-values of Paired *t*-test).

From the first two panels of Table 2, we can see that our methods CR and CRSTAR achieve higher accuracy (AUC values) than other methods. The paired *t*-test further shows that the performance improvements are significant. This is because NoN and NoSN are flexible to allow different diseases to have different tissue-specific molecular networks while the heterogeneous network model forces all diseases to share the same molecular network. Moreover, CRSTAR achieves better performance than CR. This demonstrates the effectiveness of NoSN to integrate multiple tissue-specific molecular networks for each disease.

Note that CIPHER-DN is generally worse than CIPHER-SP. In the following, we consider CIPHER-SP as the representative of CIPHER algorithm, which is also the focus of the original work [8]. We also evaluate the baseline methods using a generic PPIN which was used to generate the tissue-specific PPINs [16]. The results can be found in the Additional file 2 (Sec. More Results on Accuracy Evaluation). The results demonstrate that these methods perform better on the tissue-specific PPINs than on the generic PPIN in terms of prediction accuracy.

### Robustness evaluation

Next, we evaluate whether the selected methods are robust to noises. In our tissue-specific GCNs, we set a threshold on the gene expression levels to select genes for each tissue. This threshold controls the qualities of the resulted GCNs. To evaluate the robustness of the selected methods, we use the datasets in the previous subsection and vary the thresholds for constructing the tissue-specific GCNs. Note that in this subsection GCNs (instead of PPINs) are used as the tissue-specific molecular networks in the heterogeneous network model and NoN, and center networks in NoSN.

**Fig. 3:**
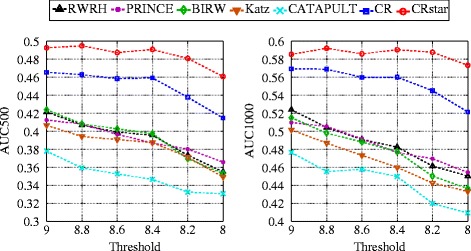
Robustness evaluation. The threshold is set to select tissue-specific genes to construct tissue-specific GCNs

The average expression value in the GCNs is around 7. Figure 3 shows the AUC500 and AUC1000 of the selected methods when varying the threshold between 8 and 9. A higher threshold keeps genes that are more tissue-specific, and a lower threshold introduces more noises. We omit the performance of CIPHER-SP since its AUC500 and AUC1000 values are very low.

From the results, we have the following observations. First, CRSTAR performs the best, with approximately 3 to 5 % AUC500 (1 to 6 % AUC1000) improvement over CR and 8 to 10 % AUC500 (6 to 12 % AUC1000) improvement over other methods. Both CR and CRSTAR perform better than other methods, with approximately 5 to 10 % AUC500 (5 to 12 % AUC1000) improvement. These verify their effectiveness to integrate multiple tissue-specific molecular networks. Second, all methods perform better with higher threshold (e.g., threshold value 9), which corresponds to lower noise level. Third, both CR and CRSTAR are more robust to noise than other methods, since the gaps between these two methods and the remaining ones become larger when more noises are introduced. This is because NoN and NoSN allow each disease to have its own tissue-specific molecular networks, while the heterogeneous network model uses a single molecular network thus is more sensitive to noise. Fourth, CR STAR is more robust than CR. This attributes to the capability of NoSN to integrate multiple types of molecular networks for a single disease.

We also evaluate the effects of the parameters of CR and CRSTAR. CR has two parameters *β* and *c*, CRSTAR has three parameters *α*, *β* and *c*. For both CR and CRSTAR, we fix *c*=0.85 which is a typical setting for label propagation methods and random walk with restart methods such as PageRank [36]. We test the remaining parameters of CR and CRSTAR on the datasets used in the previous subsection.

**Fig. 4:**
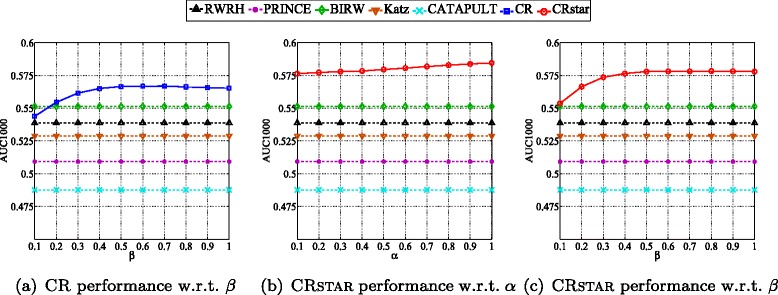
Effects of parameters on the performance of CR and CRSTAR

Figure 4 shows the effects of the parameters. AUC1000 is used as a measure of performance. For CRSTAR, we fix *β*=0.5 when varying *α* and fix *α*=0.3 when varying *β*. The performance of other methods are also presented for reference. We observe that both CR and CRSTAR are not sensitive to their parameters, their performance are stable in a large range of values of their respective *α* and *β*. In our experiments, we set *β*=0.5 for CR, *α*=0.3 and *β*=0.5 for CRSTAR.

### Automatically inferring weights of auxiliary networks

In this section, we evaluate the effectiveness of WCRSTAR. Recall that WCRSTAR allows to automatically infer the relative importances of different auxiliary networks. This is achieved by assigning lower *α*_*ip*_ values to higher ranking inconsistencies between the auxiliary networks and the center network $\Phi ^{'}_{\text {cross}}(\mathbf {r}_{i*}, \mathbf {r}_{ip})$. In this way, the more noisy auxiliary networks will contribute less than the higher quality auxiliary networks.

To generate multiple auxiliary networks for each disease, we construct another set of tissue-specific GCNs. The tissue-specific gene expression profile is obtained from [37], where 353 samples are available for 14 tissues. Using the same processing steps as before, we construct tissue-specific GCNs and assign them to their corresponding diseases.

**Fig. 5:**
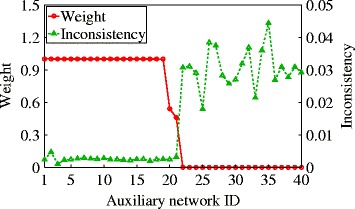
Learned weights and corresponding ranking inconsistencies

Figure 5 shows the learned auxiliary network weights *α*_*ip*_ and their corresponding ranking inconsistencies $\Phi ^{'}_{\text {cross}}(\mathbf {r}_{i*}, \mathbf {r}_{ip})$ when querying a random disease. The learned weights are sorted in decreasing order. From the figure, we can see that WCRSTAR can automatically assign low weights to high inconsistencies. In this way, it can effectively utilize the information in high quality networks and at the same is also robust to the noisy low quality networks.

The prediction accuracies of CRSTAR and WCRSTAR on this dataset are shown in the third panel of Table 2 (i.e., network model NoSN ^b^). The results of other methods are the same as before, since they do not use auxiliary networks. Comparing the performance of CRSTAR in the second and third panels, we can see that without learning the relative importances of auxiliary networks, the performance of CRSTAR does not improve with more auxiliary networks. However, WCRSTAR gives the best AUCs among all methods by learning optimal weights for auxiliary networks. This indicates that WCRSTAR can effectively leverage useful information from the additional networks.

### Evaluation on finding new associations

To evaluate the capabilities of the selected methods on predicting newly discovered associations, we apply them on the associations obtained before May 2007 to predict the associations obtained between May 2007 and May 2010. There are 439 associations before May 2007 and 126 new associations after May 2007. All other dataset settings are the same as those in Sec. Accuracy Evaluation.

**Fig. 6:**
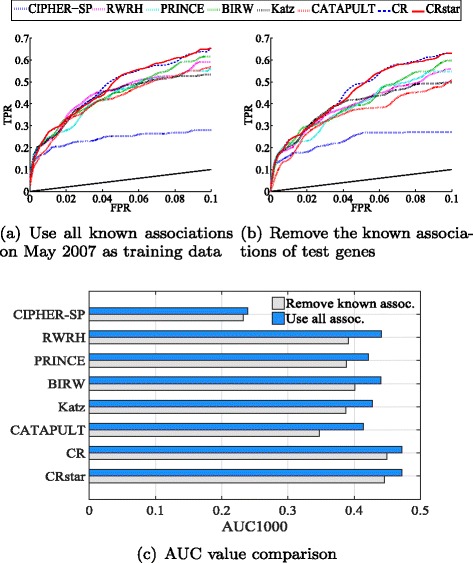
ROC curve and AUC value comparisons on predicting new associations. The *black solid lines* in **a** and **b** denote what random guess would have achieved

Figure 6(a) shows the ROC curves of the selected methods. This task is more difficult than that of cross validation since less associations are known on May 2007. Figure 6(c) (blue bars) shows the AUC1000 values of different methods in Fig. 6(a). These AUC values can be compared to the AUC1000 values in Table 2. By this comparison, we observe decreases in performance of all methods compared to the results of cross validation. Consistently, CR and CRSTAR outperform other methods.

In addition, some test genes have known associations with diseases other than the query diseases on May 2007. Such genes can be easily predicted if they are associated with diseases that are similar to the query diseases. If we remove such known associations, the results are shown in Fig. 6(b) and (c) (gray bars). As we can see from the AUC values, all methods show decreases in performance as compared with using all associations (blue bars), indicating the difficulty of this second task. On the other hand, the performance gaps between our methods and other methods in Fig. 6(b) and (c) become more obvious. This indicates that CR and CRSTAR are more effective in predicting associations involving new genes that have no known associations.

#### The importance to use NoN and NoSN

Next we give a concrete example to show that it is essential to allow diseases to have their own tissue-specific molecular networks. The association between disease *d*_1_ (OMIM record: MIM 114480) and gene ATM is known after May 2007. It is an unknown association to be discovered using the dataset before May 2007.

This association is ranked 16 by CR and CRSTAR while the highest rank given by other methods is 178 (by RWRH). The tissue associated with *d*_1_ is prostrate. *d*_1_ has two neighbors, *d*_2_ (MIM 176807) and *d*_3_ (MIM 151623), in the disease similarity network. The tissues associated with *d*_2_ and *d*_3_ are prostrate and adrenal cortex, respectively. In the May-2007 version, *d*_2_ has no known causal genes, *d*_3_ has a known causal gene TP53. In the tissue-specific PPIN of adrenal cortex, ATM is a neighbor of TP53 with high similarity 0.9999, which results in the high rank of ATM in the tissue-specific PPIN of prostrate by CR and CRSTAR. However, TP53 does not exist in the tissue-specific PPIN of prostrate. Other methods force all diseases to share the tissue-specific PPIN of prostrate, thus are not able to search ATM through the association between *d*_3_ and TP53. This shows the importance to allow different diseases to have different tissue-specific molecular networks.

## Conclusion

The existing network-based methods for disease gene prioritization often exploit a heterogeneous network model that combine prior knowledge about disease similarities, gene relationships and disease-gene associations. A major drawback of this network structure is that it forces all diseases to share the same molecular network. Recent studies demonstrate that disease genes tend to express in the tissues where the corresponding diseases manifest. In this paper, we exploit novel network models, NoN and NoSN, to model this genetic dynamics of diseases. In NoN, each disease corresponds to its most associated tissue-specific molecular network. In NoSN, each disease can have multiple tissue-specific molecular networks with complementary information. A family of ranking algorithms, CR, CRSTAR, and WCRSTAR, are developed on NoN and NoSN with rigorous theoretical analysis on their optimality and convergence properties. Extensive experimental results on real datasets from OMIM database demonstrate our methods recover true associations more accurately than other 7 popular network-based disease gene prioritization methods in terms of AUC values, with statistically significant differences (paired *t*-test *p*-values less than 0.05). The results also validate the robustness of our methods when using noisy gene co-expression networks, with approximately 5 to 12 % improvement of AUC values over the compared methods. These results confirm that our network models are flexible and effective in incorporating tissue specificities of diseases for disease gene prioritization task, and our ranking algorithms can effectively work on our novel network models.

## Endnote

^1^ http://en.wikipedia.org/wiki/Weighted_correlation_network_analysis.

## Additional files

Additional file 1 Supplementary material of WCRstar. Optimization solution to J_WCRstar_, Algorithm WCRstar and the theoretical analysis of its complexity and convergence. (PDF 117 kb)

Additional file 2 Supplementary material of experimental results. More experimental results on the accuracy evaluation, the effects of a parameter on the selectivity performance of WCRstar, and the *p*-values of paired *t*-test. (PDF 102 kb)

Additional file 3 Supplementary material of CR. Matrix form of J_CR_, optimization solution to J_CR_, Algorithm CR and the theoretical analysis of its complexity, convergence and optimality. (PDF 117 kb)

Additional file 4 Supplementary material of CRstar. Matrix form of J_CRstar_, optimization solution to J_CRstar_, Algorithm CRstar and the theoretical analysis of its complexity, convergence and optimality. (PDF 139 kb)

## Abbreviations

GCN: Gene co-expression network
NoN: Network of networks
NoSN: Network of star networks
PPIN: Protein-protein interaction network
