# Supplementary material for: Disease gene prioritization by integrating tissue-specific molecular networks using a robust multi-network model
Source: BMC Bioinformatics. 2016 Nov 10;17:453. doi: 10.1186/s12859-016-1317-x (PMC5103411; doi:10.1186/s12859-016-1317-x)
Supplement: Supplementary file 1 — Supplementary material of WCRstar. Optimization solution to JWCRstar, Algorithm WCRstar and the theoretical analysis of its complexity and convergence. (PDF 117 kb) [file 12859_2016_1317_MOESM1_ESM.pdf]

RESEARCH

[Supplementary Material] Disease gene prioritization by integrating tissue-specific molecular networks using a robust multi-network model

Jingchao Ni[1], Mehmet Koyuturk[1], Hanghang Tong[2], Jonathan Haines[3], Rong Xu[3] and Xiang Zhang[4*]

*Correspondence: xzhang@ist.psu.edu
[4] College of Information Sciences and Technology, Pennsylvania State University, 332 Information Sciences and Technology Building, PA 16802, University Park, USA
Full list of author information is available at the end of the article

Summary

In this supplementary material, we provide the optimization solution to J_{WCRstar}, the algorithm and the theoretical analysis of WCRSTAR.

Algorithm WCRstar

Algorithm 1: WCRSTAR

Input: (1) a disease similarity network $\mathbf{A}$; (2) the tissue-specific molecular networks $\{\mathbf{G}_{i*}\}$ and $\{\mathbf{G}_{ip}\}$; (3) the seed vectors $\{\mathbf{e}_{i*}\}$ and $\{\mathbf{e}_{ip}\}$; and (4) the parameters β, γ and c
Output: the ranking vectors $\{\mathbf{r}_{i*}\}$ and weights $\{\alpha_{ip}\}$

Offline-computation: Construct $\{\tilde{\mathbf{G}}_{i*}\}$, $\{\tilde{\mathbf{G}}_{ip}\}$, $\{\tilde{\mathbf{S}}_{i*,ip}\}$ and $\{\tilde{\mathbf{Y}}_{i*,j*}\}$ from $\mathbf{A}$, $\{\mathbf{G}_{i*}\}$ and $\{\mathbf{G}_{ip}\}$;
Online-ranking:
Initialize $\alpha_{ip} = 1/k_i$, $\forall i = 1, ..., h$, $p = 1, ..., k_i$;
while *not convergence* **do**
  for $i \leftarrow 1$ *to* h **do**
    Update $\mathbf{r}_{i*}$ by Eq. (1);
    for $p \leftarrow 1$ *to* k_i **do**
      Update $\mathbf{r}_{ip}$ by Eq. (2);
      $\phi_{ip} \leftarrow \Phi'_{\text{cross}}(\mathbf{r}_{i*}, \mathbf{r}_{ip})$;
    end
    Sort $\{\phi_{ip}\}_{1 \le p \le k_i}$ in increasing order;
    $t \leftarrow k_i + 1$;
    do
      $t \leftarrow t - 1$;
      $\lambda_i \leftarrow \frac{2\gamma + \sum_{p=1}^{t} \phi_{ip}}{t}$;
    while $\lambda_i - \phi_{it} \le 0$ *and* $t > 1$
    for $p \leftarrow 1$ *to* t **do**
      $\alpha_{ip} \leftarrow \frac{\lambda_i - \phi_{ip}}{2\gamma}$;
    end
    for $p \leftarrow t + 1$ *to* k_i **do**
      $\alpha_{ip} \leftarrow 0$;
    end
  end
end
return the ranking vectors $\{\mathbf{r}_{i*}\}$ and weights $\{\alpha_{ip}\}$

Complexity Analysis of WCRstar

Let n_{i*} and n_{ip} be the number of nodes in $\mathbf{G}_{i*}$ and $\mathbf{G}_{ip}$, respectively, and $n_* = \sum_{i=1}^{h} n_{i*}$, $n = \sum_{i=1}^{h}(n_{i*} + \sum_{p=1}^{k_i} n_{ip})$. Let m_{i*} and m_{ip} be the number of edges in $\mathbf{G}_{i*}$ and $\mathbf{G}_{ip}$, respectively, and $m = \sum_{i=1}^{h}(m_{i*} + \sum_{p=1}^{k_i} m_{ip})$. Let $K = \max_{1 \le i \le h} k_i$.

The offline-computation time complexity and space complexity of Algorithm 1 are the same as Algorithm CRSTAR in the Additional file 2. The online-ranking step requires $O(T^*(m + n + (h + K)n_* + hK^2))$ to update $\{\mathbf{r}_{i*}\}$, $\{\mathbf{r}_{ip}\}$, $\{\phi_{ip}\}$ and $\{\alpha_{ip}\}$ where T^* is the total number of iterations before convergence.

Generally, $n_* \le n$, h and K are much smaller than n and can be regarded as constants. Hence we can regard the time and space complexities of Algorithm 1 as $O(T^*(m + n))$ and $O(m + n)$, respectively.

Optimization Solution to $\mathrm{J}_{\mathrm{WCRstar}}$

We solve the objective function $\mathrm{J}_{\mathrm{WCRstar}}$ by an alternating minimization approach, i.e., the objective function is alternately minimized with respect to one variable while fixing others. This procedure repeats until convergence. In this section, we provide the solutions to $\mathbf{r}_{i*}$, $\mathbf{r}_{ip}$ and $\boldsymbol{\alpha}_i$. Algorithm 1 summarizes our approach according to the optimization solution.

Solutions to $\mathbf{r}_{i*}$ and $\mathbf{r}_{ip}$.

We solve $\mathbf{r}_{i*}$ and $\mathbf{r}_{ip}$ according to Theorem 1 and Theorem 2, respectively.

Theorem 1 ***Updating*** $\mathbf{r}_{i*}$. *Fixing other variables, updating* $\mathbf{r}_{i*}$ *according to Eq.* (1) *monotonically decreases the value of the objective function* $J_{WCRstar}$ *until convergence.*

$$\mathbf{r}_{i*} \leftarrow \frac{c}{\omega_i}\tilde{\mathbf{G}}_{i*}\mathbf{r}_{i*} + \frac{1-c}{\omega_i}\mathbf{e}_{i*} + \sum_{p=1}^{k_i}\frac{\alpha_{ip}}{\omega_i}\tilde{\mathbf{S}}_{i*,ip}\mathbf{r}_{ip} + \frac{2\beta}{\omega_i}\sum_{j\in\mathcal{N}_{\mathbf{A}}(i)}\tilde{\mathbf{Y}}_{i*,j*}\mathbf{r}_{j*} \tag{1}$$

where

$$\omega_i = \begin{cases} 1 + \frac{1}{k_i} + 2\beta, & \text{if } k_i \ge 1 \\ 1 + 2\beta, & \text{if } k_i = 0 \end{cases}$$

$\mathcal{N}_{\mathbf{A}}(i)$ is the neighbor set of disease i in $\mathbf{A}$, $\tilde{\mathbf{Y}}_{i*,j*} = d_{\mathbf{A}}(i)^{-\frac{1}{2}}\mathbf{A}(i,j)\mathbf{O}_{ij}d_{\mathbf{A}}(j)^{-\frac{1}{2}}$ is the $(1,1)^{\mathrm{th}}$ block of $\tilde{\mathbf{Y}}_{ij}$ and $\tilde{\mathbf{Y}}_{ij}$ is the $(i,j)^{\mathrm{th}}$ block of $\tilde{\mathbf{Y}}$.

Proof This update rule can be derived by taking the gradient descent of $\mathrm{J}_{\mathrm{WCRstar}}$ w.r.t. $\mathbf{r}_{i*}$ and setting the step size as $\frac{1}{2\omega_i}$. Its convergence can be shown in a similar way to Theorem 2 (Convergence of CR) in the Additional file 1 (Sec. Theoretical Analysis of CR) and Theorem 2 (Convergence of CRSTAR) in the Additional file 2 (Sec. Theoretical Analysis of CRSTAR) since the eigenvalues of $\tilde{\mathbf{G}}_{i*}$ are in the range of $[-1, 1]$. □

Theorem 2 ***Updating*** $\mathbf{r}_{ip}$. *Fixing other variables, updating* $\mathbf{r}_{ip}$ *according to Eq.* (2) *monotonically decreases the value of the objective function* $J_{WCRstar}$ *until convergence.*

$$\mathbf{r}_{ip} \leftarrow \frac{c}{1+\alpha_{ip}}\tilde{\mathbf{G}}_{ip}\mathbf{r}_{ip} + \frac{1-c}{1+\alpha_{ip}}\mathbf{e}_{ip} + \frac{\alpha_{ip}}{1+\alpha_{ip}}\tilde{\mathbf{S}}_{i*,ip}^{T}\mathbf{r}_{i*} \tag{2}$$

Proof This update rule can be derived by taking the gradient descent of $\mathrm{J}_{\mathrm{WCRstar}}$ w.r.t. $\mathbf{r}_{ip}$ and setting the step size as $\frac{1}{2(1+\alpha_{ip})}$. Its convergence can be shown in a similar way to Theorem 2 (Convergence of CR) in the Additional file 1 (Sec. Theoretical Analysis of CR) and Theorem 2 (Convergence of CRSTAR) in the Additional file 2 (Sec. Theoretical Analysis of CRSTAR) since the eigenvalues of $\tilde{\mathbf{G}}_{ip}$ are in the range of $[-1, 1]$. □

Solution to $\boldsymbol{\alpha}_i$.

Since we take an alternating minimization approach, we regard other variables as constants when we solve for $\boldsymbol{\alpha}_i$. Let $\boldsymbol{\phi}_i = (\Phi'_{\mathrm{cross}}(\mathbf{r}_{i*}, \mathbf{r}_{i1}), ..., \Phi'_{\mathrm{cross}}(\mathbf{r}_{i*}, \mathbf{r}_{ik_i}))^T$, we can rewrite the objective function $\mathrm{J}_{\mathrm{WCRstar}}$ w.r.t. $\boldsymbol{\alpha}_i$ by ignoring constants as

$$\begin{aligned} \min_{\boldsymbol{\alpha}_i,\ 1\le i\le h} & \mathrm{J}_{\mathrm{WCRstar}}(\boldsymbol{\alpha}_i) = \sum_{i=1}^{h} (\boldsymbol{\alpha}_i^T \boldsymbol{\phi}_i + \gamma \boldsymbol{\alpha}_i^T \boldsymbol{\alpha}_i) \\ & \text{s.t.}\ \ \boldsymbol{\alpha}_i \ge \mathbf{0},\ \boldsymbol{\alpha}_i^T \mathbf{1} = 1 \end{aligned} \tag{3}$$

where $\mathbf{1}$ is a length k_i column vector of all ones.

Eq. (3) is a quadratic optimization problem w.r.t. $\boldsymbol{\alpha}_i$. Define the Lagrange function w.r.t. $\boldsymbol{\alpha}_1, ..., \boldsymbol{\alpha}_h$ of $\mathrm{J}_{\mathrm{WCRstar}}(\boldsymbol{\alpha}_i)$ as

$$\mathrm{L}_{\mathrm{WCRstar}}(\boldsymbol{\alpha}_1, ..., \boldsymbol{\alpha}_h, \boldsymbol{\mu}_1, ..., \boldsymbol{\mu}_h, \lambda_1, ..., \lambda_h) = \sum_{i=1}^{h} \left(\boldsymbol{\alpha}_i^T \boldsymbol{\phi}_i + \gamma \boldsymbol{\alpha}_i^T \boldsymbol{\alpha}_i - \boldsymbol{\alpha}_i^T \boldsymbol{\mu}_i - \lambda_i (\boldsymbol{\alpha}_i^T \mathbf{1} - 1) \right)$$

where $\boldsymbol{\mu}_i = (\mu_{i1}, ..., \mu_{ik_i})^T \ge 0$ and $\lambda_i \ge 0$ are Lagrange multipliers. The optimal $\boldsymbol{\alpha}_i^*$ should satisfy the following Karush-Kuhn-Tucker (KKT) conditions [1]:

(1) *Gradient condition.* $\frac{\partial \mathrm{L}_{\mathrm{WCRstar}}}{\partial \boldsymbol{\alpha}_i^*} = \boldsymbol{\phi}_i + 2\gamma \boldsymbol{\alpha}_i^* - \boldsymbol{\mu}_i - \lambda_i \mathbf{1} = \mathbf{0}$

(2) *Feasibility.* $\boldsymbol{\alpha}_i^* \ge \mathbf{0}$, $(\boldsymbol{\alpha}_i^*)^T \mathbf{1} - 1 = 0$

(3) *Complementary slackness.* $\mu_{ip} \alpha_{ip}^* = 0$, $1 \le p \le k_i$

(4) *Nonnegativity.* $\boldsymbol{\mu}_i \ge \mathbf{0}$

From the gradient condition, we can obtain α_{ip}

$$\alpha_{ip} = \frac{\mu_{ip} + \lambda_i - \phi_{ip}}{2\gamma}$$

Thus α_{ip} relies on the specification of μ_{ip} and λ_i, where we have three cases [2]:

(1) When $\lambda_i - \phi_{ip} > 0$, since $\mu_{ip} \ge 0$, we have $\alpha_{ip} > 0$. From the complementary slackness, $\mu_{ip} \alpha_{ip} = 0$, we have $\mu_{ip} = 0$ thus $\alpha_{ip} = \frac{\lambda_i - \phi_{ip}}{2\gamma}$

(2) When $\lambda_i - \phi_{ip} < 0$, since $\alpha_{ip} \ge 0$, we have $\mu_{ip} > 0$. Because $\mu_{ip} \alpha_{ip} = 0$, we have $\alpha_{ip} = 0$

(3) When $\lambda_i - \phi_{ip} = 0$, we have $\alpha_{ip} = \frac{\mu_{ip}}{2\gamma}$. Since $\mu_{ip} \alpha_{ip} = 0$, we have $\alpha_{ip} = 0$ and $\mu_{ip} = 0$

Therefore, if we sort $\phi_{i1} \le \phi_{i2} \le ... \le \phi_{ik_i}$, there can be $\lambda_i > 0$ s.t. $\lambda_i - \phi_{it} > 0$ and $\lambda_i - \phi_{it+1} \le 0$. Thus α_{ip} can be solved as

$$\alpha_{ip} = \begin{cases} \frac{\lambda_i - \phi_{ip}}{2\gamma}, & \text{if } p \le t \\ 0, & \text{otherwise} \end{cases} \tag{4}$$

where λ_i can be solved by using $\sum_{p=1}^{k_i} \alpha_{ip} = 1$

$$\lambda_i = \frac{2\gamma + \sum_{p=1}^{t} \phi_{ip}}{t} \tag{5}$$

Eq. (4) implies the intuition of weight assignments. When ϕ_{ip} is large, α_{ip} is small. Recall ϕ_{ip} is the ranking inconsistency $\Phi'_{\text{cross}}(\mathbf{r}_{i*}, \mathbf{r}_{ip})$ between the center network of disease i and its pth auxiliary network. The inconsistency may come from the noise in the auxiliary network. Thus, Eq. (4) assigns small weights to large center-auxiliary ranking inconsistencies to get a consensus ranking and improve the ranking quality.

In Eq. (5), γ relates to the selectivity of the model. When γ is very large, λ_i becomes large and all auxiliary networks will be selected with nearly equal weights. When γ is very small, at least one auxiliary network (with the smallest ϕ_{ip}) will be selected. Therefore, we can use γ to control the auxiliary network integration for the ranking.

From Eq. (4) and Eq. (5), we can search the value of t decreasingly from k_i to 1 [2]. Once $\lambda_i - \phi_{it} > 0$, we find the value of t. Then we can calculate $\alpha_{i1}, ..., \alpha_{ik_i}$ according to Eq. (4). The algorithm for solving $\{\alpha_{ip}\}$ is involved in Algorithm 1. Note in Algorithm 1, when we find t, we have $\lambda_i - \phi_{it} > 0$ and $\lambda_i - \phi_{it+1} \leq 0$. The former is obvious. The latter is because at previous iteration, $\lambda_i = \frac{2\gamma + \sum_{p=1}^{t+1} \phi_{ip}}{t+1} \leq \phi_{it+1}$, which gives $2\gamma + \sum_{p=1}^{t+1} \phi_{ip} \leq (t+1)\phi_{it+1}$. Then $2\gamma + \sum_{p=1}^{t} \phi_{ip} \leq t\phi_{it+1}$ and $\frac{2\gamma + \sum_{p=1}^{t} \phi_{ip}}{t} \leq \phi_{it+1}$. Thus $\lambda_i - \phi_{it+1} \leq 0$.

Author details
[1] Department of Electrical Engineering and Computer Science, Case Western Reserve University, 10900 Euclid Avenue, OH 44106, Cleveland, USA. [2] School of Computing, Informatics, Decision Systems Engineering, Arizona State University, 699 S. Mill Ave., AZ 85281, Tempe, USA. [3] Department of Epidemiology and Biostatistics, Case Western Reserve University, 10900 Euclid Avenue, OH 44106, Cleveland, USA. [4] College of Information Sciences and Technology, Pennsylvania State University, 332 Information Sciences and Technology Building, PA 16802, University Park, USA.

References

1. Boyd, S., Vandenberghe, L.: Convex optimization. Cambridge university press (2009)
2. Yu, G., Rangwala, H., Domeniconi, C., Zhang, G., Zhang, Z.: Protein function prediction by integrating multiple kernels. In: IJCAI, pp. 1869–1875 (2013). AAAI Press
