# Supplementary material for: Disease gene prioritization by integrating tissue-specific molecular networks using a robust multi-network model
Source: BMC Bioinformatics. 2016 Nov 10;17:453. doi: 10.1186/s12859-016-1317-x (PMC5103411; doi:10.1186/s12859-016-1317-x)
Supplement: Supplementary file 2 — Supplementary material of experimental results. More experimental results on the accuracy evaluation, the effects of a parameter on the selectivity performance of WCRstar, and the p-values of paired t-test. (PDF 102 kb) [file 12859_2016_1317_MOESM2_ESM.pdf]

RESEARCH

[Supplementary Material] Disease gene prioritization by integrating tissue-specific molecular networks using a robust multi-network model

Jingchao Ni[1], Mehmet Koyuturk[1], Hanghang Tong[2], Jonathan Haines[3], Rong Xu[3] and Xiang Zhang[4*]

*Correspondence: xzhang@ist.psu.edu
[4] College of Information Sciences and Technology, Pennsylvania State University, 332 Information Sciences and Technology Building, PA 16802, University Park, USA
Full list of author information is available at the end of the article

Summary
In this supplementary material, we provide more experimental results on the performance of the compared methods on a generic PPIN which is used to generate the tissue-specific PPINs [1], the effects of a parameter on the selectivity performance of WCRSTAR, and the p-values of paired t-test.

More Results on Accuracy Evaluation
In this section, we provide the results of the baseline methods on a generic PPIN which was used to generate tissue-specific PPINs [1]. The results are shown in Table 1, which can be compared to the results of Table 2 in the paper. Comparing the results on the tissue-specific PPINs and the generic PPIN, the performance of all baseline methods are reduced using the generic PPIN, except CIPHER-DN. This is consistent with the results in [1]. The reason for the better performance of CIPHER-DN on the generic PPIN may be that the generic PPIN is more connected than the tissue-specific PPINs, increasing the chance that CIPHER-DN successfully searches neighboring genes of known disease genes (note CIPHER-DN is a neighborhood based method [2]). However, the performance of CIPHER-DN on the generic PPIN is still not competitive.

We also provide the results of ProDiGe [3], a supervised link prediction method, in Table 2. The results in Table 2 can be compared with the results of Table 2 in the paper. The authors of [4] have shown that CATAPULT performs better than ProDiGe, this is consistent with our results. From Table 2, we observe that ProDiGe works better on the generic PPIN than on the tissue-specific PPINs. The possible reason is that ProDiGe is sensitive to the number of training examples (i.e., the number of known disease-gene associations), which is less in a tissue-specific PPIN than in the generic PPIN, since a tissue-specific PPIN contains a subset of genes of the generic PPIN which are specific to the tissue. This makes ProDiGe difficult to train a good link prediction model on the tissue-specific PPINs and results in inferior performance.

Selectivity of Parameter γ of WCRstar
As mentioned in the paper (Sec. Weighted CrossRankStar) and in the Additional file 3 (Sec. Optimization Solution to J_{WCRstar}), the parameter γ in the objective function J_{WCRstar} relates to the selectivity of Algorithm WCRSTAR. Figure

Table 1 AUC values of the baseline methods on the generic PPIN. The p-value ranges: *0.005 $\sim$ 0.05, **0.0005 $\sim$ 0.005, *$<$ 0.0005, which represent the significance of performance difference.**

Method	AUC50	AUC100	AUC300	AUC500	AUC700	AUC1000
CIPHER-DN	0.2273***	0.2460***	0.2738***	0.2896***	0.3049***	0.3350***
CIPHER-SP	0.1750***	0.2160***	0.2804***	0.3060***	0.3201***	0.3351***
RWRH	0.2175***	0.2748***	0.3578***	0.4087***	0.4425***	0.4801***
PRINCE	0.2440***	0.2886***	0.3602***	0.3983***	0.4271***	0.4616***
BIRW	0.2256***	0.2898***	0.3846***	0.4328***	0.4664***	0.5020***
Katz	0.1862***	0.2471***	0.3367***	0.3903***	0.4285***	0.4704***
CATAPULT	0.1074***	0.1598***	0.2645***	0.3183***	0.3570***	0.4050***

Table 2 AUC value of ProDiGe. ProDiGe (g) represents the results on the generic PPIN. ProDiGe (t) represents the results on the tissue-specific PPINs. The p-value ranges: *0.005 $\sim$ 0.05, **0.0005 $\sim$ 0.005, *$<$ 0.0005, which represent the significance of performance difference.**

Method	AUC50	AUC100	AUC300	AUC500	AUC700	AUC1000
ProDiGe (g)	0.2126***	0.2359***	0.2520***	0.2605***	0.2659***	0.2744***
ProDiGe (t)	0.0396***	0.0403***	0.0407***	0.0408***	0.0408***	0.0408***

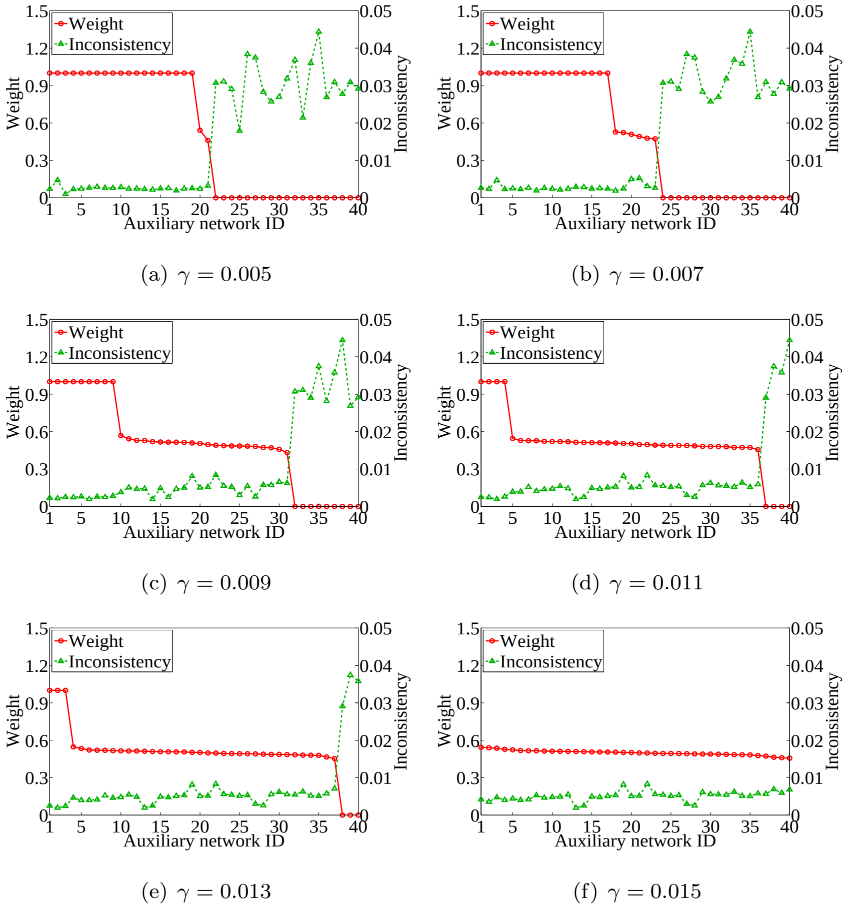

(a) $\gamma = 0.005$ (b) $\gamma = 0.007$

(c) $\gamma = 0.009$ (d) $\gamma = 0.011$

(e) $\gamma = 0.013$ (f) $\gamma = 0.015$

Figure 1 Effects of parameter γ on the selectivity of WCRstar.

1 shows the learned weights $\{\alpha_{ip}\}$ and the corresponding ranking inconsistencies $\{\Phi'_{\text{cross}}(\mathbf{r}_{i*}, \mathbf{r}_{ip})\}$ of a random validation run by various values of γ. The datasets are the same as those in the paper (Sec. Automatically Inferring Weights of Auxiliary Networks). The learned weights are sorted in decreasing order.

Table 3 **The p-values of paired t-test between the AUC values of CRstar and each other method in the first two panels of Table 2 (i.e., network models Heterogeneous network (or HN), NoN and NoSN[a]) in the paper.**

Network Model	Method	AUC50	AUC100	AUC300	AUC500	AUC700	AUC1000
HN	CIPHER-DN	$1.59e^{-04}$	$1.28e^{-07}$	$4.15e^{-14}$	$2.74e^{-18}$	$4.03e^{-22}$	$1.09e^{-25}$
	CIPHER-SP	$5.92e^{-06}$	$2.17e^{-06}$	$1.46e^{-07}$	$3.07e^{-09}$	$1.19e^{-10}$	$9.55e^{-12}$
	RWRH	$1.26e^{-04}$	$1.27e^{-05}$	$1.05e^{-04}$	$4.91e^{-03}$	$3.26e^{-03}$	$4.86e^{-03}$
	PRINCE	$2.59e^{-02}$	$8.60e^{-03}$	$7.41e^{-04}$	$1.80e^{-04}$	$3.89e^{-05}$	$1.67e^{-04}$
	BIRW	$2.46e^{-02}$	$7.30e^{-03}$	$3.66e^{-02}$	$6.01e^{-02}$	$2.50e^{-02}$	$2.06e^{-02}$
	Katz	$3.59e^{-07}$	$7.59e^{-06}$	$3.84e^{-03}$	$1.54e^{-02}$	$4.90e^{-03}$	$1.96e^{-03}$
	CATAPULT	$1.51e^{-11}$	$6.93e^{-11}$	$5.26e^{-07}$	$7.27e^{-06}$	$2.20e^{-05}$	$8.52e^{-05}$
NoN	CR	$2.75e^{-02}$	$5.46e^{-02}$	$1.40e^{-01}$	$3.01e^{-01}$	$1.80e^{-01}$	$1.44e^{-01}$
NoSN	CRSTAR	–	–	–	–	–	–

The results are consistent with the analysis in the Additional file 3 (Sec. Optimization Solution to J_{WCRstar}). The larger the value of γ, the more auxiliary networks will be selected with nearly equal weights.

The p-values of Paired t-test

The p-values of paired t-test between the AUC values of CRSTAR and each other method in the first two panels of Table 2 (i.e., network models Heterogeneous network (or HN), NoN and NoSN[a]) in the paper are summarized in Table 3. Note that each method has a vector of AUC values from the leave-one-out cross validation. Each entry in the vector comes from one validation run, that is, the AUC value for one test gene. Each time, the paired t-test is performed between the AUC vector generated by CRSTAR and the one generated by one of other methods.

Author details

[1] Department of Electrical Engineering and Computer Science, Case Western Reserve University, 10900 Euclid Avenue, OH 44106, Cleveland, USA. [2] School of Computing, Informatics, Decision Systems Engineering, Arizona State University, 699 S. Mill Ave., AZ 85281, Tempe, USA. [3] Department of Epidemiology and Biostatistics, Case Western Reserve University, 10900 Euclid Avenue, OH 44106, Cleveland, USA. [4] College of Information Sciences and Technology, Pennsylvania State University, 332 Information Sciences and Technology Building, PA 16802, University Park, USA.

References

1. Magger, O., Waldman, Y.Y., Ruppin, E., Sharan, R.: Enhancing the prioritization of disease-causing genes through tissue specific protein interaction networks. PLoS Comput. Biol. **8**(9), 1002690 (2012)
2. Wu, X., Jiang, R., Zhang, M.Q., Li, S.: Network-based global inference of human disease genes. Mol. Syst. Biol. **4**(1) (2008)
3. Mordelet, F., Vert, J.-P.: Prodige: Prioritization of disease genes with multitask machine learning from positive and unlabeled examples. BMC Bioinform. **12**(1), 389 (2011)
4. Singh-Blom, U.M., Natarajan, N., Tewari, A., Woods, J.O., Dhillon, I.S., Marcotte, E.M.: Prediction and validation of gene-disease associations using methods inspired by social network analyses. PLoS One **8**(5), 58977 (2013)
