# Supplementary material for: Disease gene prioritization by integrating tissue-specific molecular networks using a robust multi-network model
Source: BMC Bioinformatics. 2016 Nov 10;17:453. doi: 10.1186/s12859-016-1317-x (PMC5103411; doi:10.1186/s12859-016-1317-x)
Supplement: Supplementary file 3 — Supplementary material of CR. Matrix form of JCR, optimization solution to JCR, Algorithm CR and the theoretical analysis of its complexity, convergence and optimality. (PDF 117 kb) [file 12859_2016_1317_MOESM3_ESM.pdf]

RESEARCH

[Supplementary Material] Disease gene prioritization by integrating tissue-specific molecular networks using a robust multi-network model

Jingchao Ni[1], Mehmet Koyuturk[1], Hanghang Tong[2], Jonathan Haines[3], Rong Xu[3] and Xiang Zhang[4*]

*Correspondence: xzhang@ist.psu.edu
[4] College of Information Sciences and Technology, Pennsylvania State University, 332 Information Sciences and Technology Building, PA 16802, University Park, USA
Full list of author information is available at the end of the article

Summary

In this supplementary material, we provide the matrix form of $\mathrm{J_{CR}}$, the optimization solution to $\mathrm{J_{CR}}$, the algorithm and the theoretical analysis of CR.

Algorithm CR

Algorithm 1: CR

Input: (1) a disease similarity network $\mathbf{A}$; (2) the tissue-specific molecular networks $\{\mathbf{G}_i\}$; (3) the seed vectors $\{\mathbf{e}_i\}$; and (4) the parameters β and c

Output: the ranking vectors $\mathbf{r}_1, ..., \mathbf{r}_h$

```
Offline-computation: Construct G̃ and Ỹ from A and {G_i};
Online-ranking:
Construct the aggregated seed vector e = (e_1^T, ..., e_h^T)^T;
Initialize the aggregated ranking vector r = e;
while not convergence do
    Update: r ← (c/(1+2β) G̃ + 2β/(1+2β) Ỹ) r + (1−c)/(1+2β) e;
end
return the ranking vectors r_1, ..., r_h based on r
```

Complexity Analysis of CR

Let n_i be the number of nodes in $\mathbf{G}_i$ and $n = \sum_{i=1}^{h} n_i$. Let m_i be the number of edges in $\mathbf{G}_i$ and $m = \sum_{i=1}^{h} m_i$. There are $O(m + hn)$ nonzero entries in $\tilde{\mathbf{G}}$ and $\tilde{\mathbf{Y}}$ in total. Thus the offline-computation and online-ranking time complexities of Algorithm 1 are $O(m+hn)$ and $O(T^*(m+hn))$, respectively, where T^* is the total number of iterations. Since we need to store $\tilde{\mathbf{G}}$ and $\tilde{\mathbf{Y}}$, thus the space complexity is $O(m + hn)$.

Generally, h is much smaller than n and can be regarded as constants. Hence we can regard the time and space complexities of Algorithm 1 as $O(T^*(m + n))$ and $O(m + n)$, respectively.

Matrix Form of $\mathrm{J_{CR}}$

The objective function $\mathrm{J_{CR}}$ is jointly convex in $\mathbf{r}_1, ..., \mathbf{r}_h$. This can be shown by first deriving its matrix form.

Let $\mathbf{r} = (\mathbf{r}_1^T, ..., \mathbf{r}_h^T)^T$, $\mathbf{e} = (\mathbf{e}_1^T, ..., \mathbf{e}_h^T)^T$, i.e., we concatenate all ranking and seed vectors. Let $\tilde{\mathbf{G}} = \text{diag}(\tilde{\mathbf{G}}_1, ..., \tilde{\mathbf{G}}_h)$ be a diagonal block matrix. Then we have

$$c\mathbf{r}^T(\mathbf{I}_n - \tilde{\mathbf{G}})\mathbf{r} + (1-c)\|\mathbf{r} - \mathbf{e}\|_F^2 = \sum_{i=1}^{h} \Theta_{\text{within}}(\mathbf{r}_i) \tag{1}$$

where $\mathbf{I}_n$ is an $n \times n$ identity matrix and $n = \sum_{i=1}^{h} n_i$.

Define a common gene mapping matrix $\mathbf{O}_{ij} \in \{0,1\}^{n_i \times n_j}$ where $\mathbf{O}_{ij}(x,y) = 1$ if node x in $\mathbf{G}_i$ and node y in $\mathbf{G}_j$ represent the same gene; $\mathbf{O}_{ij} = 0$ otherwise. Then $\mathbf{Y}$ is a block matrix whose $(i,j)^{\text{th}}$ block is $\mathbf{A}(i,j)\mathbf{O}_{ij}$. Note that $\mathbf{A}(i,i) = 0$. Further, let $\mathbf{D}_{\mathbf{V}} = \text{diag}(d_{\mathbf{A}}(1)\mathbf{I}_{n_1}, ..., d_{\mathbf{A}}(h)\mathbf{I}_{n_h})$ be a diagonal matrix, where $d_{\mathbf{A}}(i) = \sum_{j=1}^{h} \mathbf{A}(i,j)$. We define $\mathbf{X} = \mathbf{D}_{\mathbf{V}}^{-\frac{1}{2}}(\mathbf{D}_{\mathbf{V}} - \mathbf{Y})\mathbf{D}_{\mathbf{V}}^{-\frac{1}{2}} = \mathbf{I}_n - \tilde{\mathbf{Y}}$, where $\tilde{\mathbf{Y}} = \mathbf{D}_{\mathbf{V}}^{-\frac{1}{2}}\mathbf{Y}\mathbf{D}_{\mathbf{V}}^{-\frac{1}{2}}$. We have

$$\mathbf{r}^T\mathbf{X}\mathbf{r} = \frac{1}{2}\sum_{i,j=1}^{h} \Theta_{\text{cross}}(\mathbf{r}_i, \mathbf{r}_j) \tag{2}$$

According to Eq. (1) and Eq. (2), we have the following theorem.

Theorem 1 ***Matrix Form of J_{CR}.*** *J_{CR} has the following matrix form*

$$\min_{\mathbf{r}\geq 0} J_{CR} = c\mathbf{r}^T(\mathbf{I}_n - \tilde{\mathbf{G}})\mathbf{r} + (1-c)\|\mathbf{r} - \mathbf{e}\|_F^2 + 2\beta\mathbf{r}^T\mathbf{X}\mathbf{r} \tag{3}$$

Proof The proof of Theorem 1 includes two equivalence validations:

(1) $c\mathbf{r}^T(\mathbf{I}_n - \tilde{\mathbf{G}})\mathbf{r} + (1-c)\|\mathbf{r} - \mathbf{e}\|_F^2 = \sum_{i=1}^{h} \Theta_{\text{within}}(\mathbf{r}_i)$

(2) $\mathbf{r}^T\mathbf{X}\mathbf{r} = \frac{1}{2}\sum_{i,j=1}^{h} \Theta_{\text{cross}}(\mathbf{r}_i, \mathbf{r}_j)$

Since the equivalence (1) is obvious, we only need to prove the equivalence (2).

According to the definition of $\mathbf{X}$ and $\mathbf{r}$, we have

$$\mathbf{r}^T\mathbf{X}\mathbf{r} = \mathbf{r}^T\mathbf{I}_n\mathbf{r} - \mathbf{r}^T\tilde{\mathbf{Y}}\mathbf{r} = \sum_{i=1}^{h}\mathbf{r}_i^T\mathbf{I}_{n_i}\mathbf{r}_i - \sum_{i,j=1}^{h}\mathbf{r}_i^T\tilde{\mathbf{Y}}_{ij}\mathbf{r}_j \tag{4}$$

where $\tilde{\mathbf{Y}}_{ij} \in \mathbb{R}_+^{n_i \times n_j}$ is the $(i,j)^{\text{th}}$ block of $\tilde{\mathbf{Y}}$. Note $\tilde{\mathbf{Y}}_{ii} = \mathbf{0}$ $(1 \leq i \leq h)$. Then let $(\mathbf{D}_{\mathbf{V}})_i$ be the ith diagonal block of $\mathbf{D}_{\mathbf{V}}$ and $\mathbf{Y}_{ij}$ be the $(i,j)^{\text{th}}$ block of $\mathbf{Y}$. Recall $\mathbf{Y}_{ij} = \mathbf{A}(i,j)\mathbf{O}_{ij}$. We have

$$\mathbf{r}^T\mathbf{X}\mathbf{r} = \frac{1}{2}\Big(\sum_{i=1}^{h}\frac{\mathbf{r}_i^T}{\sqrt{d_{\mathbf{A}}(i)}}(\mathbf{D}_{\mathbf{V}})_i\frac{\mathbf{r}_i}{\sqrt{d_{\mathbf{A}}(i)}} - 2\sum_{i,j=1}^{h}\frac{\mathbf{r}_i^T}{\sqrt{d_{\mathbf{A}}(i)}}\mathbf{Y}_{ij}\frac{\mathbf{r}_j}{\sqrt{d_{\mathbf{A}}(j)}} + \sum_{j=1}^{h}\frac{\mathbf{r}_j^T}{\sqrt{d_{\mathbf{A}}(j)}}(\mathbf{D}_{\mathbf{V}})_j\frac{\mathbf{r}_j}{\sqrt{d_{\mathbf{A}}(j)}}\Big)$$

Let $\mathbf{D}_{\mathbf{Y}}$ be the degree matrix of $\mathbf{Y}$ and $(\mathbf{D}_{\mathbf{Y}})_i$ be the ith diagonal block of $\mathbf{D}_{\mathbf{Y}}$. Define $\mathbf{D}_{\mathbf{Y}_{ij}}$ to be the degree matrix of $\mathbf{Y}_{ij}$ (note the nonzero diagonal values of $\mathbf{D}_{\mathbf{Y}_{ij}}$ are $\mathbf{A}(i,j)$). Then $(\mathbf{D}_{\mathbf{Y}})_i = \sum_{j=1}^{h}\mathbf{D}_{\mathbf{Y}_{ij}}$. Define $\bar{\mathbf{D}}_{\mathbf{Y}_{ij}}$ to be an $n_i \times n_i$ diagonal matrix s.t. $\mathbf{D}_{\mathbf{Y}_{ij}} + \bar{\mathbf{D}}_{\mathbf{Y}_{ij}} = \mathbf{A}(i,j)\mathbf{I}_{n_i}$. Then let $(\bar{\mathbf{D}}_{\mathbf{Y}})_i = \sum_{j=1}^{h}\bar{\mathbf{D}}_{\mathbf{Y}_{ij}}$, we

have $(\mathbf{D_V})_i = (\mathbf{D_Y} + \bar{\mathbf{D}}_\mathbf{Y})_i$. Thus

$$\begin{aligned}
\mathbf{r}^T\mathbf{X}\mathbf{r} &= \frac{1}{2}\Big(\sum_{i=1}^{h}\frac{\mathbf{r}_i^T}{\sqrt{d_\mathbf{A}(i)}}(\mathbf{D_Y}+\bar{\mathbf{D}}_\mathbf{Y})_i\frac{\mathbf{r}_i}{\sqrt{d_\mathbf{A}(i)}} - 2\sum_{i,j=1}^{h}\frac{\mathbf{r}_i^T}{\sqrt{d_\mathbf{A}(i)}}\mathbf{Y}_{ij}\frac{\mathbf{r}_j}{\sqrt{d_\mathbf{A}(j)}}\\
&+\sum_{j=1}^{h}\frac{\mathbf{r}_j^T}{\sqrt{d_\mathbf{A}(j)}}(\mathbf{D_Y}+\bar{\mathbf{D}}_\mathbf{Y})_j\frac{\mathbf{r}_j}{\sqrt{d_\mathbf{A}(j)}}\Big)\\
&= \frac{1}{2}\Big(\sum_{i,j=1}^{h}\frac{\mathbf{r}_i^T}{\sqrt{d_\mathbf{A}(i)}}\mathbf{D}_{\mathbf{Y}_{ij}}\frac{\mathbf{r}_i}{\sqrt{d_\mathbf{A}(i)}} - 2\sum_{i,j=1}^{h}\frac{\mathbf{r}_i^T}{\sqrt{d_\mathbf{A}(i)}}\mathbf{Y}_{ij}\frac{\mathbf{r}_j}{\sqrt{d_\mathbf{A}(j)}}\\
&+\sum_{j,i=1}^{h}\frac{\mathbf{r}_j^T}{\sqrt{d_\mathbf{A}(j)}}\mathbf{D}_{\mathbf{Y}_{ji}}\frac{\mathbf{r}_j}{\sqrt{d_\mathbf{A}(j)}} + \sum_{i,j=1}^{h}\frac{\mathbf{r}_i^T}{\sqrt{d_\mathbf{A}(i)}}\bar{\mathbf{D}}_{\mathbf{Y}_{ij}}\frac{\mathbf{r}_i}{\sqrt{d_\mathbf{A}(i)}} + \sum_{j,i=1}^{h}\frac{\mathbf{r}_j^T}{\sqrt{d_\mathbf{A}(j)}}\bar{\mathbf{D}}_{\mathbf{Y}_{ji}}\frac{\mathbf{r}_j}{\sqrt{d_\mathbf{A}(j)}}\Big)\\
&= \frac{1}{2}\sum_{i,j=1}^{h}\mathbf{A}(i,j)\Big(\frac{\mathbf{r}_i^T(\mathcal{I}_{ij})}{\sqrt{d_\mathbf{A}(i)}}\frac{\mathbf{r}_i(\mathcal{I}_{ij})}{\sqrt{d_\mathbf{A}(i)}} - 2\frac{\mathbf{r}_i^T(\mathcal{I}_{ij})}{\sqrt{d_\mathbf{A}(i)}}\frac{\mathbf{r}_j(\mathcal{I}_{ij})}{\sqrt{d_\mathbf{A}(j)}} + \frac{\mathbf{r}_j^T(\mathcal{I}_{ij})}{\sqrt{d_\mathbf{A}(j)}}\frac{\mathbf{r}_j(\mathcal{I}_{ij})}{\sqrt{d_\mathbf{A}(j)}}\\
&+\frac{\mathbf{r}_i^T(\bar{\mathcal{I}}_{ij})}{\sqrt{d_\mathbf{A}(i)}}\frac{\mathbf{r}_i(\bar{\mathcal{I}}_{ij})}{\sqrt{d_\mathbf{A}(i)}} + \frac{\mathbf{r}_j^T(\bar{\mathcal{I}}_{ji})}{\sqrt{d_\mathbf{A}(j)}}\frac{\mathbf{r}_j(\bar{\mathcal{I}}_{ji})}{\sqrt{d_\mathbf{A}(j)}}\Big)\\
&= \frac{1}{2}\sum_{i,j=1}^{h}\mathbf{A}(i,j)\Big(\|\frac{\mathbf{r}_i(\mathcal{I}_{ij})}{\sqrt{d_\mathbf{A}(i)}} - \frac{\mathbf{r}_j(\mathcal{I}_{ij})}{\sqrt{d_\mathbf{A}(j)}}\|_F^2 + \|\frac{\mathbf{r}_i(\bar{\mathcal{I}}_{ij})}{\sqrt{d_\mathbf{A}(i)}}\|_F^2 + \|\frac{\mathbf{r}_j(\bar{\mathcal{I}}_{ji})}{\sqrt{d_\mathbf{A}(j)}}\|_F^2\Big)\\
&= \frac{1}{2}\sum_{i,j=1}^{h}\Theta_{\text{cross}}(\mathbf{r}_i,\mathbf{r}_j)
\end{aligned}$$

This completes the proof. □

Optimization Solution to J_{CR}

From Theorem 1, J_{CR} is a quadratic function of $\mathbf{r}$. We can derive a power method to minimize J_{CR} as follows.

$$\frac{\partial \mathrm{J}_{\mathrm{CR}}}{\partial \mathbf{r}} = 2\Big((1+2\beta)\mathbf{I}_n - (c\tilde{\mathbf{G}} + 2\beta\tilde{\mathbf{Y}})\Big)\mathbf{r} - 2(1-c)\mathbf{e}$$

Using gradient descent, if we set $\mathbf{r} \leftarrow \mathbf{r} - \eta\frac{\partial \mathrm{J}_{\mathrm{CR}}}{\partial \mathbf{r}}$, where $\eta = \frac{1}{2(1+2\beta)}$, we have

$$\mathbf{r} \leftarrow \Big(\frac{c}{1+2\beta}\tilde{\mathbf{G}} + \frac{2\beta}{1+2\beta}\tilde{\mathbf{Y}}\Big)\mathbf{r} + \frac{1-c}{1+2\beta}\mathbf{e} \qquad (5)$$

Eq. (5) is a fixed-point approach to compute $\mathbf{r}$ that converges to the global optimal solution of J_{CR}. Algorithm 1 summarizes our approach according to the optimization solution.

Theoretical Analysis of CR

In this section, we show that Algorithm 1 converges to the global minimum of J_{CR} by Theorem 2 and Theorem 3.

Theorem 2 ***Convergence of* CR.** *Algorithm 1 converges to the closed-form solution*

$$\mathbf{r} = (\mathbf{I}_n - \frac{c}{1+2\beta}\tilde{\mathbf{G}} - \frac{2\beta}{1+2\beta}\tilde{\mathbf{Y}})^{-1}\frac{1-c}{1+2\beta}\mathbf{e}$$

Proof First, the closed-form solution can be obtained by solving $\frac{\partial \mathrm{J_{CR}}}{\partial \mathbf{r}} = 0$. Then let $\mathbf{M} = \frac{c}{1+2\beta}\tilde{\mathbf{G}} + \frac{2\beta}{1+2\beta}\tilde{\mathbf{Y}}$, the CR updating rule in Eq. (5) becomes $\mathbf{r} = \mathbf{M}\mathbf{r} + \frac{1-c}{1+2\beta}\mathbf{e}$. Next, we show that the eigenvalues of $\mathbf{M}$ are in the range of $(-1, 1)$.

Let $\mathbf{G} = \mathrm{diag}(\mathbf{G}_1, ..., \mathbf{G}_h)$ and $\mathbf{D_G}$ be its degree matrix, then $\tilde{\mathbf{G}} = \mathbf{D}_{\mathbf{G}}^{-\frac{1}{2}}\mathbf{G}\mathbf{D}_{\mathbf{G}}^{-\frac{1}{2}}$. Since $\tilde{\mathbf{G}}$ is similar to the stochastic matrix $\mathbf{G}\mathbf{D}_{\mathbf{G}}^{-1} = \mathbf{D}_{\mathbf{G}}^{\frac{1}{2}}\tilde{\mathbf{G}}\mathbf{D}_{\mathbf{G}}^{-\frac{1}{2}}$, it has eigenvalues within $[-1, 1]$. Also, $\tilde{\mathbf{Y}}$ is similar to the matrix $\mathbf{Y}\mathbf{D}_{\mathbf{V}}^{-1} = \mathbf{D}_{\mathbf{V}}^{\frac{1}{2}}\tilde{\mathbf{Y}}\mathbf{D}_{\mathbf{V}}^{-\frac{1}{2}}$ where each column sum of $\mathbf{Y}\mathbf{D}_{\mathbf{V}}^{-1}$ is no greater than 1.

The Gershgorin Circle Theorem [1] states that for a complex $n \times n$ matrix $\mathbf{B}$, every eigenvalue λ of $\mathbf{B}$ lies within at least one of the Gershgorin discs $\{\lambda : |\lambda - b_{ii}| \leq \sum_{j=1, j\neq i}^{n} |b_{ji}|\}$ $(i = 1, ..., n)$, where b_{ii} is the ith diagonal value of $\mathbf{B}$ and b_{ji} is the $(j, i)^{\text{th}}$ entry of $\mathbf{B}$. Since $\mathbf{A}(i, i) = 0$ for $i = 1, ..., h$, the diagonal values of $\mathbf{Y}$ are zero. Therefore, the eigenvalues of $\mathbf{Y}\mathbf{D}_{\mathbf{V}}^{-1}$ satisfy $|\lambda| \leq 1$, which implies the eigenvalues of $\tilde{\mathbf{Y}}$ are within $[-1, 1]$.

One result of the Weyl's Inequality Theorem [2] states that for matrices $\hat{\mathbf{H}}, \mathbf{H}, \mathbf{P} \in \mathcal{H}_n$, where $\mathcal{H}_n$ is the set of $n \times n$ Hermitian matrices, if $\hat{\mathbf{H}} = \mathbf{H} + \mathbf{P}$ and their eigenvalues are arranged in non-increasing orders, i.e., $\lambda_1(\hat{\mathbf{H}}) \geq ... \geq \lambda_n(\hat{\mathbf{H}})$, $\lambda_1(\mathbf{H}) \geq ... \geq \lambda_n(\mathbf{H})$, $\lambda_1(\mathbf{P}) \geq ... \geq \lambda_n(\mathbf{P})$, then the following inequalities hold:

$$\lambda_n(\mathbf{P}) \leq \lambda_i(\hat{\mathbf{H}}) - \lambda_i(\mathbf{H}) \leq \lambda_1(\mathbf{P}), \forall i = 1, ..., n$$

Since $\tilde{\mathbf{G}}, \tilde{\mathbf{Y}}, \mathbf{M} \in \mathcal{H}_n$ and $\mathbf{M} = \frac{c}{1+2\beta}\tilde{\mathbf{G}} + \frac{2\beta}{1+2\beta}\tilde{\mathbf{Y}}$, we have

$$\lambda_1(\mathbf{M}) \leq \frac{c}{1+2\beta}\lambda_1(\tilde{\mathbf{G}}) + \frac{2\beta}{1+2\beta}\lambda_1(\tilde{\mathbf{Y}})$$
$$\lambda_n(\mathbf{M}) \geq \frac{c}{1+2\beta}\lambda_n(\tilde{\mathbf{G}}) + \frac{2\beta}{1+2\beta}\lambda_n(\tilde{\mathbf{Y}})$$

which means the eigenvalues of $\mathbf{M}$ are in the range of $[-\frac{c+2\beta}{1+2\beta}, \frac{c+2\beta}{1+2\beta}]$. Since $0 < c < 1$, the eigenvalues of $\mathbf{M}$ are in the range of $(-1, 1)$.

Based on this property, we can show the convergence of the fixed-point approach. Without loss of generality, let $\mathbf{r}^{(0)} = \mathbf{e}$, and t be the iteration index $(t \geq 1)$. According to the CR updating rule in Eq. (5), we have

$$\mathbf{r}^{(t)} = \mathbf{M}^t\mathbf{e} + \sum_{i=0}^{t-1} \mathbf{M}^i \frac{1-c}{1+2\beta}\mathbf{e}$$

Since the eigenvalues of $\mathbf{M}$ are all in $(-1, 1)$, we have

$$\lim_{t\to\infty} \mathbf{M}^t = 0, \text{ and } \lim_{t\to\infty} \sum_{i=0}^{t-1} \mathbf{M}^i = (\mathbf{I}_n - \mathbf{M})^{-1}$$

Therefore

$$\mathbf{r} = \lim_{t\to\infty} \mathbf{r}^{(t)} = (\mathbf{I}_n - \mathbf{M})^{-1}\frac{1-c}{1+2\beta}\mathbf{e} = (\mathbf{I}_n - \frac{c}{1+2\beta}\tilde{\mathbf{G}} - \frac{2\beta}{1+2\beta}\tilde{\mathbf{Y}})^{-1}\frac{1-c}{1+2\beta}\mathbf{e}$$

which is the closed-form solution. □

Theorem 3 ***Optimality of*** **CR.** *At convergence, Algorithm 1 gives the global minimum of J_{CR} defined in Eq.* (3).

Proof This can be proved by showing that the function in Eq. (3) is convex. The Hessian matrix of Eq. (3) is $\nabla^2 \mathrm{J}_{\mathrm{CR}} = 2((1+2\beta)\mathbf{I}_n - (c\tilde{\mathbf{G}} + 2\beta\tilde{\mathbf{Y}}))$. Following the similar idea as in the proof of Theorem 2, we have that the eigenvalues of $\nabla^2 \mathrm{J}_{\mathrm{CR}}$ are no less than $2(1-c)$. Since $0 < c < 1$, $\nabla^2 \mathrm{J}_{\mathrm{CR}}$ is positive-definite. Therefore, Eq. (3) is convex. □

Author details
[1] Department of Electrical Engineering and Computer Science, Case Western Reserve University, 10900 Euclid Avenue, OH 44106, Cleveland, USA. [2] School of Computing, Informatics, Decision Systems Engineering, Arizona State University, 699 S. Mill Ave., AZ 85281, Tempe, USA. [3] Department of Epidemiology and Biostatistics, Case Western Reserve University, 10900 Euclid Avenue, OH 44106, Cleveland, USA. [4] College of Information Sciences and Technology, Pennsylvania State University, 332 Information Sciences and Technology Building, PA 16802, University Park, USA.

References

1. Varga, R.S.: Geršgorin and his circles. Springer-Verlag, Berlin (2004)
2. Bhatia, R.: Linear algebra to quantum cohomology: the story of alfred horn's inequalities. Amer. Math. Monthly, 289–318 (2001)
