# Supplementary material for: Disease gene prioritization by integrating tissue-specific molecular networks using a robust multi-network model
Source: BMC Bioinformatics. 2016 Nov 10;17:453. doi: 10.1186/s12859-016-1317-x (PMC5103411; doi:10.1186/s12859-016-1317-x)
Supplement: Supplementary file 4 — Supplementary material of CRstar. Matrix form of JCRstar, optimization solution to JCRstar, Algorithm CRstar and the theoretical analysis of its complexity, convergence and optimality. (PDF 139 kb) [file 12859_2016_1317_MOESM4_ESM.pdf]

RESEARCH

[Supplementary Material] Disease gene prioritization by integrating tissue-specific molecular networks using a robust multi-network model

Jingchao Ni[1], Mehmet Koyuturk[1], Hanghang Tong[2], Jonathan Haines[3], Rong Xu[3] and Xiang Zhang[4*]

[*]Correspondence:
xzhang@ist.psu.edu
[4] College of Information Sciences and Technology, Pennsylvania State University, 332 Information Sciences and Technology Building, PA 16802, University Park, USA
Full list of author information is available at the end of the article

Summary

In this supplementary material, we provide the matrix form of $\mathrm{J}_{\mathrm{CRstar}}$, the optimization solution to $\mathrm{J}_{\mathrm{CRstar}}$, the algorithm and the theoretical analysis of CRSTAR.

Algorithm CRstar

Algorithm 1: CRSTAR

Input: (1) a disease similarity network $\mathbf{A}$; (2) the tissue-specific molecular networks $\{\mathbf{G}_{i*}\}$ and $\{\mathbf{G}_{ip}\}$; (3) the seed vectors $\{\mathbf{e}_{i*}\}$ and $\{\mathbf{e}_{ip}\}$; and (4) the parameters α, β and c
Output: the ranking vectors $\mathbf{r}_{1*}, ..., \mathbf{r}_{h*}$

Offline-computation: Construct $\tilde{\mathbf{G}}$, $\tilde{\mathbf{S}}$ and $\tilde{\mathbf{Y}}$ from $\mathbf{A}$, $\{\mathbf{G}_{i*}\}$ and $\{\mathbf{G}_{ip}\}$;
Online-ranking:
Construct the aggregated seed vector $\mathbf{e}$ from $\{\mathbf{e}_{i*}\}$ and $\{\mathbf{e}_{ip}\}$;
Initialize the aggregated ranking vector $\mathbf{r} = \mathbf{e}$;
while *not convergence* **do**
  Update: $\mathbf{r} \leftarrow (\frac{c}{1+\alpha+2\beta}\tilde{\mathbf{G}} + \frac{\alpha}{1+\alpha+2\beta}\tilde{\mathbf{S}} + \frac{2\beta}{1+\alpha+2\beta}\tilde{\mathbf{Y}})\mathbf{r} + \frac{1-c}{1+\alpha+2\beta}\mathbf{e}$;
end
return the ranking vectors $\mathbf{r}_{1*}, ..., \mathbf{r}_{h*}$ based on $\mathbf{r}$

Complexity Analysis of CRstar

Let n_{i*} and n_{ip} be the number of nodes in $\mathbf{G}_{i*}$ and $\mathbf{G}_{ip}$, respectively, and $n_* = \sum_{i=1}^{h} n_{i*}$, $n = \sum_{i=1}^{h}(n_{i*} + \sum_{p=1}^{k_i} n_{ip})$. Let m_{i*} and m_{ip} be the number of edges in $\mathbf{G}_{i*}$ and $\mathbf{G}_{ip}$, respectively, and $m = \sum_{i=1}^{h}(m_{i*} + \sum_{p=1}^{k_i} m_{ip})$. Let $K = \max_{1\leq i\leq h} k_i$.

There are $O(m + n + (h + K)n_*)$ nonzero entries in $\tilde{\mathbf{G}}$, $\tilde{\mathbf{S}}$ and $\tilde{\mathbf{Y}}$ in total. Thus the offline-computation and online-ranking time complexities of Algorithm 1 are $O(m + n + (h + K)n_*)$ and $O(T^*(m + n + (h + K)n_*))$, respectively, where T^* is the total number of iterations. Since we need to store $\tilde{\mathbf{G}}$, $\tilde{\mathbf{S}}$ and $\tilde{\mathbf{Y}}$, thus the space complexity is $O(m + n + (h + K)n_*)$.

Generally, $n_* \leq n$, h and K are much smaller than n and can be regarded as constants. Hence we can regard the time and space complexities of Algorithm 1 as $O(T^*(m + n))$ and $O(m + n)$, respectively.

Matrix Form of $\mathrm{J_{CRstar}}$

Similar to $\mathrm{J_{CR}}$, the objective function $\mathrm{J_{CRstar}}$ is jointly convex in $\{\mathbf{r}_{i*}\}$ and $\{\mathbf{r}_{ip}\}$. To show this, we first derive its matrix form. In this section, we denote $n_i = n_{i*} + \sum_{p=1}^{k_i} n_{ip}$ and $n = \sum_{i=1}^{h} n_i$.

Let $\mathbf{r}_i = (\mathbf{r}_{i*}^T, \mathbf{r}_{i1}^T, ..., \mathbf{r}_{ik_i}^T)^T$, $\mathbf{r} = (\mathbf{r}_1^T, ..., \mathbf{r}_h^T)^T$, $\mathbf{e}_i = (\mathbf{e}_{i*}^T, \mathbf{e}_{i1}^T, ..., \mathbf{e}_{ik_i}^T)^T$, $\mathbf{e} = (\mathbf{e}_1^T, ..., \mathbf{e}_h^T)^T$, i.e., we concatenate all ranking and seed vectors. Let $\tilde{\mathbf{G}}_i = \mathrm{diag}(\tilde{\mathbf{G}}_{i*}, \tilde{\mathbf{G}}_{i1}, ..., \tilde{\mathbf{G}}_{ik_i})$, $\tilde{\mathbf{G}} = \mathrm{diag}(\tilde{\mathbf{G}}_1, ..., \tilde{\mathbf{G}}_h)$. Then the first term of $\mathrm{J_{CRstar}}$ is equivalent to

$$c\mathbf{r}^T(\mathbf{I}_n - \tilde{\mathbf{G}})\mathbf{r} + (1-c)\|\mathbf{r} - \mathbf{e}\|_F^2 \tag{1}$$

where $\mathbf{I}_n$ is an $n \times n$ identiy matrix.

Define $\mathbf{S}_{i*,ip} \in \{0,1\}^{n_{i*} \times n_{ip}}$ to be a mapping matrix of the common genes between $\mathbf{G}_{i*}$ and $\mathbf{G}_{ip}$. That is, $\mathbf{S}_{i*,ip}(x,y) = 1$ if node x in $\mathbf{G}_{i*}$ and node y in $\mathbf{G}_{ip}$ represent the same gene; $\mathbf{S}_{i*,ip}(x,y) = 0$ otherwise. Then $\mathbf{S}_i \in \{0,1\}^{n_i \times n_i}$ is defined as

$$\mathbf{S}_i = \begin{cases} \begin{bmatrix} \mathbf{0} & \mathbf{S}_{i*,i1} & ... & \mathbf{S}_{i*,ik_i} \\ \mathbf{S}_{i1,i*} & \mathbf{0} & \mathbf{0} & \mathbf{0} \\ ... & ... & ... & ... \\ \mathbf{S}_{ik_i,i*} & \mathbf{0} & \mathbf{0} & \mathbf{0} \end{bmatrix}, & \text{if } k_i \geq 1 \\ \mathbf{I}_{n_{i*}}, & \text{if } k_i = 0 \end{cases}$$

Then we let $\mathbf{S} = \mathrm{diag}(\mathbf{S}_1, ..., \mathbf{S}_h)$. Define a diagonal matrix $\mathbf{D}_{\mathbf{U}_i} = \mathrm{diag}(k_i\mathbf{I}_{n_{i*}}, \mathbf{I}_{n_{i1}}, ..., \mathbf{I}_{n_{ik_i}})$ if $k_i \geq 1$; $\mathbf{D}_{\mathbf{U}_i} = \mathbf{I}_{n_{i*}}$ if $k_i = 0$. Then $\mathbf{D}_{\mathbf{U}} = \mathrm{diag}(\mathbf{D}_{\mathbf{U}_1}, ..., \mathbf{D}_{\mathbf{U}_h})$ is a diagonal matrix. Define $\mathbf{L} = \mathbf{D}_{\mathbf{U}}^{-\frac{1}{2}}(\mathbf{D}_{\mathbf{U}} - \mathbf{S})\mathbf{D}_{\mathbf{U}}^{-\frac{1}{2}} = \mathbf{I}_n - \tilde{\mathbf{S}}$, where $\tilde{\mathbf{S}} = \mathbf{D}_{\mathbf{U}}^{-\frac{1}{2}}\mathbf{S}\mathbf{D}_{\mathbf{U}}^{-\frac{1}{2}}$. We have

$$\mathbf{r}^T\mathbf{L}\mathbf{r} = \sum_{i=1}^{h}\sum_{p=1}^{k_i} \Phi'_{\mathrm{cross}}(\mathbf{r}_{i*}, \mathbf{r}_{ip}) \tag{2}$$

For the third term of $\mathrm{J_{CRstar}}$, let $\mathbf{O}_{ij} \in \{0,1\}^{n_{i*} \times n_{j*}}$ be a common gene mapping matrix between $\mathbf{G}_{i*}$ and $\mathbf{G}_{j*}$ where $\mathbf{O}_{ij}(x,y) = 1$ if node x in $\mathbf{G}_{i*}$ and node y in $\mathbf{G}_{j*}$ represent the same gene; $\mathbf{O}_{ij}(x,y) = 0$ otherwise. Then we define $\mathbf{Y}_{ij} \in \mathbb{R}_+^{n_i \times n_j}$ as

$$\mathbf{Y}_{ij} = \begin{cases} \begin{bmatrix} \mathbf{A}(i,j)\mathbf{O}_{ij} & \mathbf{0} & ... & \mathbf{0} \\ \mathbf{0} & \mathbf{0} & \mathbf{0} & \mathbf{0} \\ ... & ... & ... & ... \\ \mathbf{0} & \mathbf{0} & \mathbf{0} & \mathbf{0} \end{bmatrix}, & \text{if } i \neq j \\ \mathrm{diag}(\mathbf{0}_{n_{i*}}, \mathbf{I}_{n_{i1}}, ..., \mathbf{I}_{n_{ik_i}}), & \text{if } i = j \end{cases}$$

and $\mathbf{Y} \in \mathbb{R}_+^{n \times n}$ is defined as a block matrix whose $(i,j)^{\mathrm{th}}$ block is $\mathbf{Y}_{ij}$. Define a diagonal matrix $\mathbf{D}_{\mathbf{V}_i} = \mathrm{diag}(d_{\mathbf{A}}(i)\mathbf{I}_{n_{i*}}, \mathbf{I}_{n_{i1}}, ..., \mathbf{I}_{n_{ik_i}})$. Then $\mathbf{D}_{\mathbf{V}} = \mathrm{diag}(\mathbf{D}_{\mathbf{V}_1}, ..., \mathbf{D}_{\mathbf{V}_h})$ is a diagonal matrix. Finally, let $\mathbf{X} = \mathbf{D}_{\mathbf{V}}^{-\frac{1}{2}}(\mathbf{D}_{\mathbf{V}} - \mathbf{Y})\mathbf{D}_{\mathbf{V}}^{-\frac{1}{2}} = \mathbf{I}_n - \tilde{\mathbf{Y}}$, where $\tilde{\mathbf{Y}} = \mathbf{D}_{\mathbf{V}}^{-\frac{1}{2}}\mathbf{Y}\mathbf{D}_{\mathbf{V}}^{-\frac{1}{2}}$. We have

$$\mathbf{r}^T\mathbf{X}\mathbf{r} = \frac{1}{2}\sum_{i,j=1}^{h} \Phi_{\mathrm{cross}}(\mathbf{r}_{i*}, \mathbf{r}_{j*}) \tag{3}$$

According to Eq. (1), Eq. (2) and Eq. (3), we have the following theorem.

Theorem 1 ***Matrix Form of J_{CRstar}.*** *J_{CRstar} has the following matrix form*

$$\min_{\mathbf{r}\geq 0} J_{CRstar} = c\mathbf{r}^T(\mathbf{I}_n - \tilde{\mathbf{G}})\mathbf{r} + (1-c)\|\mathbf{r}-\mathbf{e}\|_F^2 + \alpha\mathbf{r}^T\mathbf{L}\mathbf{r} + 2\beta\mathbf{r}^T\mathbf{X}\mathbf{r} \quad (4)$$

Proof The proof of Theorem 1 includes three equivalence validations:

(1) $c\mathbf{r}^T(\mathbf{I}_n - \tilde{\mathbf{G}})\mathbf{r} + (1-c)\|\mathbf{r}-\mathbf{e}\|_F^2 = \sum_{i=1}^h \left(\Phi_{\text{within}}(\mathbf{r}_{i*}) + \sum_{p=1}^{k_i} \Phi_{\text{within}}(\mathbf{r}_{ip})\right)$

(2) $\mathbf{r}^T\mathbf{L}\mathbf{r} = \sum_{i=1}^h \sum_{p=1}^{k_i} \Phi'_{\text{cross}}(\mathbf{r}_{i*}, \mathbf{r}_{ip})$

(3) $\mathbf{r}^T\mathbf{X}\mathbf{r} = \frac{1}{2}\sum_{i,j=1}^h \Phi_{\text{cross}}(\mathbf{r}_{i*}, \mathbf{r}_{j*})$

Since the equivalence (1) is obvious, we will prove the equivalences (2) and (3), respectively.

For the **equivalence (2)**, since $\mathbf{L}$ is a diagonal block matrix, we have $\mathbf{r}^T\mathbf{L}\mathbf{r} = \sum_{i=1}^h \mathbf{r}_i^T\mathbf{L}_i\mathbf{r}_i$ where $\mathbf{L}_i$ is the ith diagonal block of $\mathbf{L}$. We only need to show

$$\mathbf{r}_i^T\mathbf{L}_i\mathbf{r}_i = \sum_{p=1}^{k_i} \Phi'_{\text{cross}}(\mathbf{r}_{i*}, \mathbf{r}_{ip})$$

First, if $k_i = 0$, then $\mathbf{S}_i = \mathbf{I}_{n_{i*}}$ and $\mathbf{D}_{\mathbf{U}_i} = \mathbf{I}_{n_{i*}}$ according to the definition. This implies $\tilde{\mathbf{S}}_i = \mathbf{I}_{n_{i*}}$. Thus $\mathbf{r}_i^T\mathbf{L}_i\mathbf{r}_i = \mathbf{r}_i^T(\mathbf{I}_{n_{i*}} - \mathbf{I}_{n_{i*}})\mathbf{r}_i = 0$, which is equivalent to $\sum_{p=1}^{k_i} \Phi'_{\text{cross}}(\mathbf{r}_{i*}, \mathbf{r}_{ip})$ for $k_i = 0$.

If $k_i \geq 1$, for simplicity of notation, we regard the subscript $i*$ as $i1$ and increase all other subscripts by 1, then we have

$$\mathbf{r}_i^T\mathbf{L}_i\mathbf{r}_i = \sum_{p=1}^{k_i+1} \mathbf{r}_{ip}^T\mathbf{I}_{n_{ip}}\mathbf{r}_{ip} - \sum_{p,q=1}^{k_i+1} \mathbf{r}_{ip}^T\tilde{\mathbf{S}}_{ip,iq}\mathbf{r}_{iq}$$

where $\tilde{\mathbf{S}}_{ip,iq}$ is the $(p,q)^{\text{th}}$ block of $\tilde{\mathbf{S}}_i$ and $\tilde{\mathbf{S}}_i$ is the ith diagonal block of $\tilde{\mathbf{S}}$. Note $\tilde{\mathbf{S}}_{ip,iq} \neq \mathbf{0}$ if $p = 1$ or $q = 1$ but $p \neq q$. Let $\mathbf{D}_{\mathbf{U}_{ip}}$ $(1 \leq p \leq k_i + 1)$ be the pth diagonal block of $\mathbf{D}_{\mathbf{U}_i}$, $d_{\mathbf{U}_{ip}}$ be the diagonal value of $\mathbf{D}_{\mathbf{U}_{ip}}$ (recall $\mathbf{D}_{\mathbf{U}_{ip}}$ has the same diagonal values). Then

$$\mathbf{r}_i^T\mathbf{L}_i\mathbf{r}_i = \frac{1}{2}\Big(\sum_{p=1}^{k_i+1} \frac{\mathbf{r}_{ip}^T}{\sqrt{d_{\mathbf{U}_{ip}}}}\mathbf{D}_{\mathbf{U}_{ip}}\frac{\mathbf{r}_{ip}}{\sqrt{d_{\mathbf{U}_{ip}}}} - 2\sum_{p,q=1}^{k_i+1} \frac{\mathbf{r}_{ip}^T}{\sqrt{d_{\mathbf{U}_{ip}}}}\mathbf{S}_{ip,iq}\frac{\mathbf{r}_{iq}}{\sqrt{d_{\mathbf{U}_{iq}}}} + \sum_{q=1}^{k_i+1} \frac{\mathbf{r}_{iq}^T}{\sqrt{d_{\mathbf{U}_{iq}}}}\mathbf{D}_{\mathbf{U}_{iq}}\frac{\mathbf{r}_{iq}}{\sqrt{d_{\mathbf{U}_{iq}}}}\Big)$$

Let $\mathbf{D}_{\mathbf{S}_i}$ be the degree matrix of $\mathbf{S}_i$ and $\mathbf{D}_{\mathbf{S}_{ip}}$ be the pth diagonal block of $\mathbf{D}_{\mathbf{S}_i}$. Define $\mathbf{D}_{\mathbf{S}_{ip,iq}}$ to be the degree matrix of $\mathbf{S}_{ip,iq}$ (note the nonzero diagonal values of $\mathbf{D}_{\mathbf{S}_{ip,iq}}$ are 1). Then $\mathbf{D}_{\mathbf{S}_{ip}} = \sum_{q=1}^{k_i+1} \mathbf{D}_{\mathbf{S}_{ip,iq}}$. Define $\bar{\mathbf{D}}_{\mathbf{S}_{ip,iq}}$ be an $n_{ip} \times n_{ip}$ diagonal matrix s.t. $\mathbf{D}_{\mathbf{S}_{ip,iq}} + \bar{\mathbf{D}}_{\mathbf{S}_{ip,iq}} = \mathbf{I}_{n_{ip}}$ if $p = 1$ or $q = 1$ but $p \neq q$; $\bar{\mathbf{D}}_{\mathbf{S}_{ip,iq}} = \mathbf{0}$ otherwise.

Then let $\bar{\mathbf{D}}_{\mathbf{S}_{ip}} = \sum_{q=1}^{k_i+1} \bar{\mathbf{D}}_{\mathbf{S}_{ip,iq}}$, we have $\mathbf{D}_{\mathbf{U}_{ip}} = \mathbf{D}_{\mathbf{S}_{ip}} + \bar{\mathbf{D}}_{\mathbf{S}_{ip}}$. Thus

$$
\begin{aligned}
\mathbf{r}_i^T \mathbf{L}_i \mathbf{r}_i &= \frac{1}{2}\Big(\sum_{p=1}^{k_i+1} \frac{\mathbf{r}_{ip}^T}{\sqrt{d_{\mathbf{U}_{ip}}}}(\mathbf{D}_{\mathbf{S}_{ip}} + \bar{\mathbf{D}}_{\mathbf{S}_{ip}})\frac{\mathbf{r}_{ip}}{\sqrt{d_{\mathbf{U}_{ip}}}} - 2\sum_{p,q=1}^{k_i+1} \frac{\mathbf{r}_{ip}^T}{\sqrt{d_{\mathbf{U}_{ip}}}}\mathbf{S}_{ip,iq}\frac{\mathbf{r}_{iq}}{\sqrt{d_{\mathbf{U}_{iq}}}} \\
&+ \sum_{q=1}^{k_i+1} \frac{\mathbf{r}_{iq}^T}{\sqrt{d_{\mathbf{U}_{iq}}}}(\mathbf{D}_{\mathbf{S}_{iq}} + \bar{\mathbf{D}}_{\mathbf{S}_{iq}})\frac{\mathbf{r}_{iq}}{\sqrt{d_{\mathbf{U}_{iq}}}}\Big) \\
&= \frac{1}{2}\Big(\sum_{p=1}^{k_i+1} \frac{\mathbf{r}_{ip}^T}{\sqrt{d_{\mathbf{U}_{ip}}}}\mathbf{D}_{\mathbf{S}_{ip}}\frac{\mathbf{r}_{ip}}{\sqrt{d_{\mathbf{U}_{ip}}}} - 2\sum_{p,q=1}^{k_i+1} \frac{\mathbf{r}_{ip}^T}{\sqrt{d_{\mathbf{U}_{ip}}}}\mathbf{S}_{ip,iq}\frac{\mathbf{r}_{iq}}{\sqrt{d_{\mathbf{U}_{iq}}}} + \sum_{q=1}^{k_i+1} \frac{\mathbf{r}_{iq}^T}{\sqrt{d_{\mathbf{U}_{iq}}}}\mathbf{D}_{\mathbf{S}_{iq}}\frac{\mathbf{r}_{iq}}{\sqrt{d_{\mathbf{U}_{iq}}}} \\
&+ 2\sum_{p=1}^{k_i+1} \frac{\mathbf{r}_{ip}^T}{\sqrt{d_{\mathbf{U}_{ip}}}}\bar{\mathbf{D}}_{\mathbf{S}_{ip}}\frac{\mathbf{r}_{ip}}{\sqrt{d_{\mathbf{U}_{ip}}}}\Big) \\
&= \frac{1}{2}\Big(\sum_{p,q=1}^{k_i+1} \frac{\mathbf{r}_{ip}^T}{\sqrt{d_{\mathbf{U}_{ip}}}}\mathbf{D}_{\mathbf{S}_{ip,iq}}\frac{\mathbf{r}_{ip}}{\sqrt{d_{\mathbf{U}_{ip}}}} - 2\sum_{p,q=1}^{k_i+1} \frac{\mathbf{r}_{ip}^T}{\sqrt{d_{\mathbf{U}_{ip}}}}\mathbf{S}_{ip,iq}\frac{\mathbf{r}_{iq}}{\sqrt{d_{\mathbf{U}_{iq}}}} + \sum_{q,p=1}^{k_i+1} \frac{\mathbf{r}_{iq}^T}{\sqrt{d_{\mathbf{U}_{iq}}}}\mathbf{D}_{\mathbf{S}_{iq,ip}}\frac{\mathbf{r}_{iq}}{\sqrt{d_{\mathbf{U}_{iq}}}} \\
&+ 2\sum_{p,q=1}^{k_i+1} \frac{\mathbf{r}_{ip}^T}{\sqrt{d_{\mathbf{U}_{ip}}}}\bar{\mathbf{D}}_{\mathbf{S}_{ip,iq}}\frac{\mathbf{r}_{ip}}{\sqrt{d_{\mathbf{U}_{ip}}}}\Big) \\
&= \frac{1}{2}\sum_{p,q=1}^{k_i+1}\Big(\frac{\mathbf{r}_{ip}^T(\mathcal{I}_{ip,iq})}{\sqrt{d_{\mathbf{U}_{ip}}}}\frac{\mathbf{r}_{ip}(\mathcal{I}_{ip,iq})}{\sqrt{d_{\mathbf{U}_{ip}}}} - 2\frac{\mathbf{r}_{ip}^T(\mathcal{I}_{ip,iq})}{\sqrt{d_{\mathbf{U}_{ip}}}}\frac{\mathbf{r}_{iq}(\mathcal{I}_{ip,iq})}{\sqrt{d_{\mathbf{U}_{iq}}}} + \frac{\mathbf{r}_{iq}^T(\mathcal{I}_{ip,iq})}{\sqrt{d_{\mathbf{U}_{iq}}}}\frac{\mathbf{r}_{iq}(\mathcal{I}_{ip,iq})}{\sqrt{d_{\mathbf{U}_{iq}}}} \\
&+ 2\frac{\mathbf{r}_{ip}^T(\bar{\mathcal{I}}_{ip,iq})}{\sqrt{d_{\mathbf{U}_{ip}}}}\frac{\mathbf{r}_{ip}(\bar{\mathcal{I}}_{ip,iq})}{\sqrt{d_{\mathbf{U}_{ip}}}}\Big) \\
&= \frac{1}{2}\sum_{p,q=1}^{k_i+1}\Big(\|\frac{\mathbf{r}_{ip}(\mathcal{I}_{ip,iq})}{\sqrt{d_{\mathbf{U}_{ip}}}} - \frac{\mathbf{r}_{iq}(\mathcal{I}_{ip,iq})}{\sqrt{d_{\mathbf{U}_{iq}}}}\|_F^2 + 2\|\frac{\mathbf{r}_{ip}(\bar{\mathcal{I}}_{ip,iq})}{\sqrt{d_{\mathbf{U}_{ip}}}}\|_F^2\Big)
\end{aligned}
$$

According to the definitions of $\mathbf{S}_i$, $\mathbf{D}_{\mathbf{S}_{ip,iq}}$ and $\bar{\mathbf{D}}_{\mathbf{S}_{ip,iq}}$, $\mathcal{I}_{ip,iq} \neq \emptyset$ and $\bar{\mathcal{I}}_{ip,iq} \neq \emptyset$ if $p = 1$ or $q = 1$ but $p \neq q$. According to the definition of $\mathbf{D}_{\mathbf{U}_i}$, $d_{\mathbf{U}_{ip}} = k_i$ if $p = 1$ and $d_{\mathbf{U}_{ip}} = 1$ if $2 \leq p \leq k_i + 1$. Therefore

$$
\begin{aligned}
\mathbf{r}_i^T \mathbf{L}_i \mathbf{r}_i &= \frac{1}{2}\sum_{q=2}^{k_i+1}\Big(\|\frac{\mathbf{r}_{i1}(\mathcal{I}_{i1,iq})}{\sqrt{k_i}} - \mathbf{r}_{iq}(\mathcal{I}_{i1,iq})\|_F^2 + 2\|\frac{\mathbf{r}_{i1}(\bar{\mathcal{I}}_{i1,iq})}{\sqrt{k_i}}\|_F^2\Big) \\
&+ \frac{1}{2}\sum_{p=2}^{k_i+1}\Big(\|\mathbf{r}_{ip}(\mathcal{I}_{ip,i1}) - \frac{\mathbf{r}_{i1}(\mathcal{I}_{ip,i1})}{\sqrt{k_i}}\|_F^2 + 2\|\mathbf{r}_{ip}(\bar{\mathcal{I}}_{ip,i1})\|_F^2\Big) \\
&= \sum_{p=2}^{k_i+1}\Big(\|\frac{\mathbf{r}_{i1}(\mathcal{I}_{i1,ip})}{\sqrt{k_i}} - \mathbf{r}_{ip}(\mathcal{I}_{i1,ip})\|_F^2 + \|\frac{\mathbf{r}_{i1}(\bar{\mathcal{I}}_{i1,ip})}{\sqrt{k_i}}\|_F^2 + \|\mathbf{r}_{ip}(\bar{\mathcal{I}}_{ip,i1})\|_F^2\Big)
\end{aligned}
$$

Replace subscript 1 to $*$, decrease other subscripts by 1, we have

$$
\mathbf{r}_i^T \mathbf{L}_i \mathbf{r}_i = \sum_{p=1}^{k_i} \Phi'_{\mathrm{cross}}(\mathbf{r}_{i*}, \mathbf{r}_{ip})
$$

For the **equivalence (3)**, according to the definition of $\mathbf{X}$, we can consider $\mathbf{X}$ as a block matrix with $\mathbf{X}_{ij} \in \mathbb{R}^{n_i \times n_j}$ $(1 \leq i, j \leq h)$ corresponding to each $\mathbf{Y}_{ij}$ as

$$
\mathbf{X}_{ij} = \begin{cases} \begin{bmatrix} -\tilde{\mathbf{Y}}_{i*,j*} & \mathbf{0} & ... & \mathbf{0} \\ \mathbf{0} & \mathbf{0} & \mathbf{0} & \mathbf{0} \\ ... & ... & ... & ... \\ \mathbf{0} & \mathbf{0} & \mathbf{0} & \mathbf{0} \end{bmatrix}, & \text{if } i \neq j \\ \mathrm{diag}(\mathbf{I}_{n_{i*}}, \mathbf{0}_{n_{i1}}, ..., \mathbf{0}_{n_{ik_i}}), & \text{if } i = j \end{cases}
$$

where $\tilde{\mathbf{Y}}_{i*,j*} = d_{\mathbf{A}}(i)^{-\frac{1}{2}}\mathbf{A}(i,j)\mathbf{O}_{ij}d_{\mathbf{A}}(j)^{-\frac{1}{2}}$ is the $(1,1)^{\text{th}}$ block of $\tilde{\mathbf{Y}}_{ij}$ and $\tilde{\mathbf{Y}}_{ij}$ is the $(i,j)^{\text{th}}$ block of $\tilde{\mathbf{Y}}$. Since only the first diagonal block of $\mathbf{X}_{ij}$ is nonzero, we have

$$\mathbf{r}^T\mathbf{X}\mathbf{r} = \sum_{i=1}^{h}\mathbf{r}_{i*}^T\mathbf{I}_{n_{i*}}\mathbf{r}_{i*} - \sum_{i,j=1}^{h}\mathbf{r}_{i*}^T\tilde{\mathbf{Y}}_{i*,j*}\mathbf{r}_{j*} \tag{5}$$

Note $\tilde{\mathbf{Y}}_{i*,i*} = \mathbf{0}$ $(1 \le i \le h)$. Since Eq. (5) is equivalent to Eq. (4) in the proof of Theorem 1 (Matrix Form of J_{CR}) in the Additional file 1 (Sec. Matrix Form of J_{CR}), the proof for equivalence (3) is similar to the proof of Theorem 1 in the Additional file 1 thus omitted.

Combining equivalences (1), (2), (3), we complete the proof of Theorem 1. □

Optimization Solution to $\mathrm{J}_{\mathrm{CRstar}}$

From Theorem 1, $\mathrm{J}_{\mathrm{CRstar}}$ is a quadratic function of $\mathbf{r}$. We can derive a power method to minimize $\mathrm{J}_{\mathrm{CRstar}}$ as follows.

$$\frac{\partial \mathrm{J}_{\mathrm{CRstar}}}{\partial \mathbf{r}} = 2\Big((1+\alpha+2\beta)\mathbf{I}_n - (c\tilde{\mathbf{G}} + \alpha\tilde{\mathbf{S}} + 2\beta\tilde{\mathbf{Y}})\Big)\mathbf{r} - 2(1-c)\mathbf{e}$$

Using gradient descent, if we set $\mathbf{r} \leftarrow \mathbf{r} - \eta\frac{\partial \mathrm{J}}{\partial \mathbf{r}}$, where $\eta = \frac{1}{2(1+\alpha+2\beta)}$, we have

$$\mathbf{r} \leftarrow \Big(\frac{c}{\omega}\tilde{\mathbf{G}} + \frac{\alpha}{\omega}\tilde{\mathbf{S}} + \frac{2\beta}{\omega}\tilde{\mathbf{Y}}\Big)\mathbf{r} + \frac{1-c}{\omega}\mathbf{e} \tag{6}$$

where $\omega = 1+\alpha+2\beta$. Eq. (6) is a fixed-point approach to compute $\mathbf{r}$ that converges to the global optimal solution of $\mathrm{J}_{\mathrm{CRstar}}$. Algorithm 1 summarizes our approach according to the optimization solution.

Theoretical Analysis of CRstar

In this section, we show that Algorithm 1 converges to the global minimum of $\mathrm{J}_{\mathrm{CRstar}}$ by Theorem 2 and Theorem 3.

Theorem 2 ***Convergence of* CRstar.** *Algorithm 1 converges to the closed-form solution*

$$\mathbf{r} = (\mathbf{I}_n - \frac{c}{\omega}\tilde{\mathbf{G}} - \frac{\alpha}{\omega}\tilde{\mathbf{S}} - \frac{2\beta}{\omega}\tilde{\mathbf{Y}})^{-1}\frac{1-c}{\omega}\mathbf{e}$$

where $\omega = 1+\alpha+2\beta$.

Proof First, the closed-form solution can be obtained by solving $\frac{\partial \mathrm{J}_{\mathrm{CRstar}}}{\partial \mathbf{r}} = 0$. Then let $\mathbf{M} = \frac{c}{\omega}\tilde{\mathbf{G}} + \frac{\alpha}{\omega}\tilde{\mathbf{S}} + \frac{2\beta}{\omega}\tilde{\mathbf{Y}}$, the CRSTAR updating rule in Eq. (6) becomes $\mathbf{r} = \mathbf{M}\mathbf{r} + \frac{1-c}{\omega}\mathbf{e}$. Next, we show that the eigenvalues of $\mathbf{M}$ are in the range of $(-1,1)$.

Let $\mathbf{G}_i = \mathrm{diag}(\mathbf{G}_{i*}, \mathbf{G}_{i1}, ..., \mathbf{G}_{ik_i})$, $\mathbf{G} = \mathrm{diag}(\mathbf{G}_1, ..., \mathbf{G}_h)$ and $\mathbf{D}_{\mathbf{G}}$ be the degree matrix of $\mathbf{G}$, then $\tilde{\mathbf{G}} = \mathbf{D}_{\mathbf{G}}^{-\frac{1}{2}}\mathbf{G}\mathbf{D}_{\mathbf{G}}^{-\frac{1}{2}}$. Since $\tilde{\mathbf{G}}$ is similar to the stochastic matrix $\mathbf{G}\mathbf{D}_{\mathbf{G}}^{-1} = \mathbf{D}_{\mathbf{G}}^{\frac{1}{2}}\tilde{\mathbf{G}}\mathbf{D}_{\mathbf{G}}^{-\frac{1}{2}}$, it has eigenvalues within $[-1,1]$. Also, $\tilde{\mathbf{S}}$ and $\tilde{\mathbf{Y}}$ are similar to the matrices $\mathbf{S}\mathbf{D}_{\mathbf{U}}^{-1} = \mathbf{D}_{\mathbf{U}}^{\frac{1}{2}}\tilde{\mathbf{S}}\mathbf{D}_{\mathbf{U}}^{-\frac{1}{2}}$ and $\mathbf{Y}\mathbf{D}_{\mathbf{V}}^{-1} = \mathbf{D}_{\mathbf{V}}^{\frac{1}{2}}\tilde{\mathbf{Y}}\mathbf{D}_{\mathbf{V}}^{-\frac{1}{2}}$, respectively, where each column sum of $\mathbf{S}\mathbf{D}_{\mathbf{U}}^{-1}$ and $\mathbf{Y}\mathbf{D}_{\mathbf{V}}^{-1}$ is no greater than 1. Therefore, both $\tilde{\mathbf{S}}$ and $\tilde{\mathbf{Y}}$ have eigenvalues within $[-1,1]$ according to the Gershgorin Circle Theorem [1].

Since $\tilde{\mathbf{G}}, \tilde{\mathbf{S}}, \tilde{\mathbf{Y}}, \mathbf{M} \in \mathcal{H}_n$ and $\mathbf{M} = \frac{c}{\omega}\tilde{\mathbf{G}} + \frac{\alpha}{\omega}\tilde{\mathbf{S}} + \frac{2\beta}{\omega}\tilde{\mathbf{Y}}$, where $\omega = 1 + \alpha + 2\beta$, according to the Weyl's Inequality Theorem [2], we have

$$\lambda_1(\mathbf{M}) \leq \frac{c}{\omega}\lambda_1(\tilde{\mathbf{G}}) + \frac{\alpha}{\omega}\lambda_1(\tilde{\mathbf{S}}) + \frac{2\beta}{\omega}\lambda_1(\tilde{\mathbf{Y}})$$
$$\lambda_n(\mathbf{M}) \geq \frac{c}{\omega}\lambda_n(\tilde{\mathbf{G}}) + \frac{\alpha}{\omega}\lambda_n(\tilde{\mathbf{S}}) + \frac{2\beta}{\omega}\lambda_n(\tilde{\mathbf{Y}})$$

which means the eigenvalues of $\mathbf{M}$ are in the range of $[-\frac{c+\alpha+2\beta}{1+\alpha+2\beta}, \frac{c+\alpha+2\beta}{1+\alpha+2\beta}]$. Since $0 < c < 1$, the eigenvalues of $\mathbf{M}$ are in the range of $(-1, 1)$.

Based on this property, we can show the convergence of the fixed-point approach in a similar way to the proof of Theorem 2 (Convergence of CR) in the Additional file 1 (Sec. Theoretical Analysis of CR) by letting $\mathbf{r}^{(0)} = \mathbf{e}$ and showing

$$\mathbf{r} = \lim_{t\to\infty} \mathbf{r}^{(t)} = (\mathbf{I}_n - \mathbf{M})^{-1}\frac{1-c}{\omega}\mathbf{e} = (\mathbf{I}_n - \frac{c}{\omega}\tilde{\mathbf{G}} - \frac{\alpha}{\omega}\tilde{\mathbf{S}} - \frac{2\beta}{\omega}\tilde{\mathbf{Y}})^{-1}\frac{1-c}{\omega}\mathbf{e}$$

where t is the iteration index ($t \geq 1$). This completes the proof. □

Theorem 3 ***Optimality of*** **CRstar.** *At convergence, Algorithm 1 gives the global minimum of J_{CRstar} defined in Eq.* (4)

Proof This can be proved by showing that the function in Eq. (4) is convex. The Hessian matrix of Eq. (4) is $\nabla^2 \mathrm{J}_{\mathrm{CRstar}} = 2((1 + \alpha + 2\beta)\mathbf{I}_n - (c\tilde{\mathbf{G}} + \alpha\tilde{\mathbf{S}} + 2\beta\tilde{\mathbf{Y}}))$. Following the similar idea as in the proof of Theorem 2, we have that the eigenvalues of $\nabla^2 \mathrm{J}_{\mathrm{CRstar}}$ are no less than $2(1-c)$. Since $0 < c < 1$, $\nabla^2 \mathrm{J}_{\mathrm{CRstar}}$ is positive-definite. Therefore, Eq. (4) is convex. □

Author details
[1] Department of Electrical Engineering and Computer Science, Case Western Reserve University, 10900 Euclid Avenue, OH 44106, Cleveland, USA. [2] School of Computing, Informatics, Decision Systems Engineering, Arizona State University, 699 S. Mill Ave., AZ 85281, Tempe, USA. [3] Department of Epidemiology and Biostatistics, Case Western Reserve University, 10900 Euclid Avenue, OH 44106, Cleveland, USA. [4] College of Information Sciences and Technology, Pennsylvania State University, 332 Information Sciences and Technology Building, PA 16802, University Park, USA.

References
1. Varga, R.S.: Geršgorin and his circles. Springer-Verlag, Berlin (2004)
2. Bhatia, R.: Linear algebra to quantum cohomology: the story of alfred horn's inequalities. Amer. Math. Monthly, 289–318 (2001)
